# A murine model of large-scale bone regeneration reveals a selective requirement for *Sonic Hedgehog*

**DOI:** 10.1038/s41536-022-00225-8

**Published:** 2022-05-17

**Authors:** Maxwell A. Serowoky, Stephanie T. Kuwahara, Shuwan Liu, Venus Vakhshori, Jay R. Lieberman, Francesca V. Mariani

**Affiliations:** 1 Department of Stem Cell Biology and Regenerative Medicine, Keck School of Medicine, University of Southern California, 1425 San Pablo Street, Los Angeles, CA 90089 USA; 2 Department of Orthopaedic Surgery, Keck School of Medicine, University of Southern California, 1520 San Pablo Street, Los Angeles, CA 90089 USA

**Keywords:** Regeneration, Mesenchymal stem cells

## Abstract

Building and maintaining skeletal tissue requires the activity of skeletal stem and progenitor cells (SSPCs). Following injury, local pools of these SSPCs become active and coordinate to build new cartilage and bone tissues. While recent studies have identified specific markers for these SSPCs, how they become activated in different injury contexts is not well-understood. Here, using a model of large-scale rib bone regeneration in mice, we demonstrate that the growth factor, Sonic Hedgehog (SHH), is an early and essential driver of large-scale bone healing. *Shh* expression is broadly upregulated in the first few days following rib bone resection, and conditional knockout of *Shh* at early but not late post-injury stages severely inhibits cartilage callus formation and later bone regeneration. Whereas Smoothened (*Smo*), a key transmembrane component of the Hh pathway, is required in Sox9+ lineage cells for rib regeneration, we find that *Shh* is required in a *Prrx1-expressing, Sox9-*negative mesenchymal population. Intriguingly, upregulation of *Shh* expression and requirements for *Shh* and *Smo* may be unique to large-scale injuries, as they are dispensable for both complete rib and femur fracture repair. In addition, single-cell RNA sequencing of callus tissue from animals with deficient Hedgehog signaling reveals a depletion of *Cxcl12*-expressing cells, which may indicate failed recruitment of *Cxcl12*-expressing SSPCs during the regenerative response. These results reveal a mechanism by which *Shh* expression in the local injury environment unleashes large-scale regenerative abilities in the murine rib.

## Introduction

Some animals such as salamanders, zebrafish, and lizards are famously endowed with the ability to regenerate exceptionally large and complex skeletal structures, however humans and other mammalian vertebrates are typically only capable of healing minor skeletal injuries. For example, bone lesions in humans >1 cm^3^ are generally defined as “critical-sized” defects that will not repair without surgical intervention^1^. A notable exception is the human rib cage, which is capable of regenerating astonishingly large skeletal segments^2^. Inspired by case-reports of large-scale rib regeneration in adult humans^3–5^, our lab recently discovered that ribs of adult mice are similarly capable of regenerating unusually large skeletal segments^2,6,7^. Using this novel model, we identified the periosteum as a key source of specialized skeletal stem/progenitor cells (SSPCs) that orchestrate large-scale bone regeneration^2,6,7^. Additionally, we have shown that *Sox9-*expressing periosteal SSPCs require the Hedgehog (Hh) signaling pathway component Smoothened (*Smo*) for proper differentiation during the first few days of large-scale regeneration^7^. These results are in stark contrast to recent data showing that inhibition of Hh signaling does not prevent fracture healing^8,9^, suggesting that the scale of the injury may be a major component that determines the requirement for Hedgehog signaling.

It is now appreciated that skeletal regeneration is executed by a combination of multiple SSPC populations found locally within the periosteum, bone marrow (BM), and skeletal muscle interstitial compartments^10–16^. For example, a recent report highlights the importance of BM-derived *Cxcl12*+ stromal cells for generating chondrocytes during bone repair^13^. Following injury, most long bones regenerate through endochondral ossification whereby a cartilage callus stabilizes and reduces the fracture prior to the conversion of the cartilage callus to bone^17^. The earliest steps of injury-induced activation of SSPCs and their subsequent differentiation into a cartilage callus are not completely understood. It is clear that SSPCs from multiple tissues generate callus chondrocytes during repair^7,15,16,18^; however, it is unknown how cells within these compartments coordinate their activities in different injury contexts.

Here, we show that Sonic Hedgehog (*Shh*) is required for the full repair of large-scale bone injuries in the rib, but not small-scale fractures in mice. We find that *Shh* expression is rapidly and broadly upregulated at the site of injury in response to large-scale injury and that conditional knockout of *Shh* severely inhibits regeneration. Hedgehog signaling is especially critical during the earliest stages of regeneration, as conditional genetic knockout of *Shh* or *Smo* beginning at 5 days post injury does not affect subsequent healing. In addition, we observed a decrease in the number of *Cxcl12*+ cells in the central region of the callus of *Smo* cKO mice, which may reflect impaired recruitment of BM-derived *Cxcl12*+ SSPCs. Together, these data shed light on the selective expression and requirement of *Shh* during large-scale regeneration.

## Results

### *Shh* is expressed during early callus formation

**Fig. 1:**
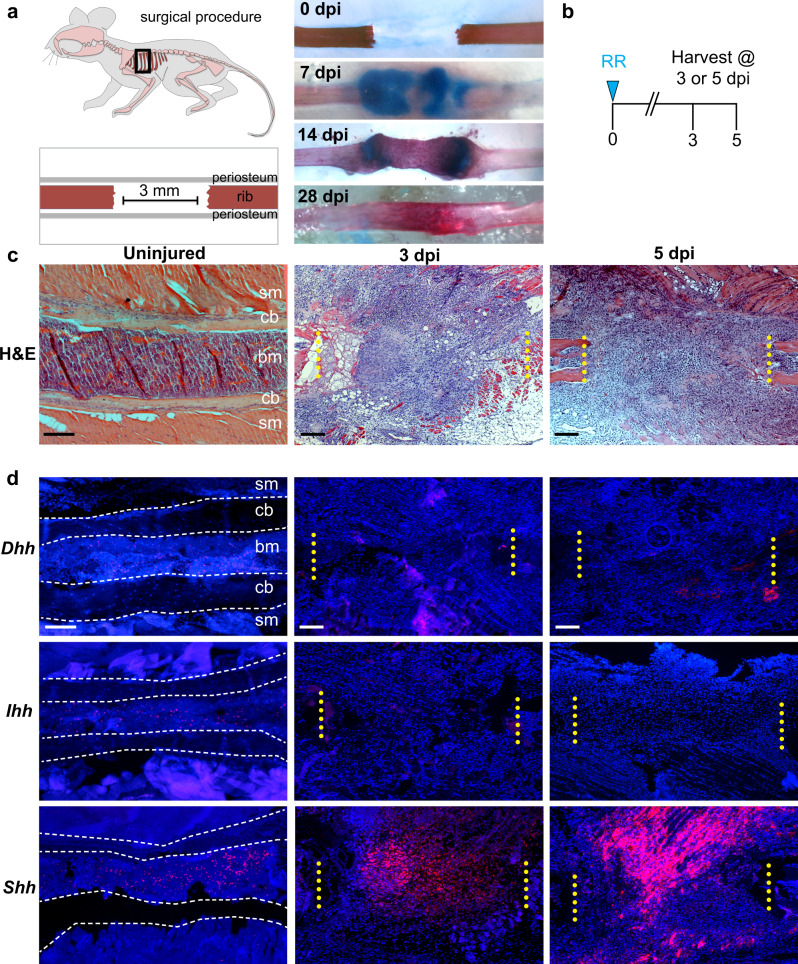
*Shh*, but not *Dhh* or *Ihh* is upregulated during early callus formation. **a** Overview of resection procedure. Briefly, a 3 mm segmental resection of rib bone is performed leaving adjacent periosteal and muscle tissues intact. Whole-mount samples are stained with alizarin red and alcian blue as indicators of mineralized bone and cartilage respectively. 0 days post injury (dpi) shows the empty resection gap. By 7 dpi, alcian blue stained cartilage bridges the gap. At 14 dpi, the cartilage callus has converted to alizarin red stained bone with some alcian blue cartilage remaining adjacent to cut sites. By 28 dpi, the callus has substantially remodeled back to the original anatomy. **b** Experimental overview. Rib resections of C57BL/6J control mice were performed at day 0 and tissues were harvested at 3 or 5 dpi for analysis in **c**. **c** Hematoxylin and eosin (H&E) staining of uninjured, 3 dpi, and 5 dpi repair calluses shows mesenchymal cells filling the resection gap prior to chondrogenesis which typically begins at 7 dpi. sm = skeletal muscle, cb = cortical bone, bm = bone marrow. Surgical cut sites are indicated with yellow dotted lines. Scale bars: Uninjured = 100 microns, 3 dpi and 5 dpi = 200 microns. **d** RNA-ISH for *Dhh*, *Ihh*, and *Shh* on histological sections harvested from an uninjured animal or at 3 dpi and 5 dpi. In uninjured animals, *Dhh* and *Ihh* are detectable at low levels in the bone marrow and in a few cells at 3 and 5 dpi. *Shh* is detectable at low levels in uninjured animals in the bone marrow but not in mature osteocytes, periosteal tissue, or adjacent skeletal muscle. *N* ≥ 2. At 3 dpi, *Shh* is strongly detected within cells located in a central region of the resection gap and modestly upregulated in the surrounding periosteal and skeletal muscle tissues, *N* = 3. At 5 dpi the central domain of *Shh* expression has expanded and intensified, *N* = 5. sm = skeletal muscle, cb = cortical bone, bm = bone marrow. Scale bars: Uninjured = 100 microns, 3 dpi and 5 dpi = 200 microns.

Our previous work identified a population of periosteal resident SSPCs marked with a *Sox9:*CreER transgene that requires the Hedgehog signaling component *Smoothened* (*Smo*) for proper large-scale rib regeneration^7^. The Hh ligands that are responsible for activating *Sox9*+ SSPCs remain unclear. We therefore set out to characterize the expression of the three known Hedgehog ligands, *Desert Hedgehog (Dhh), Indian Hedgehog (Ihh)*, and *Sonic Hedgehog (Shh)*, during the early response to large-scale rib resection. Briefly, this injury model includes surgical resection of a 3 mm segment of rib bone while leaving adjacent periosteal and muscle tissues intact (Fig. 1a). Repair of this injury model in control mice results in the formation a large cartilage callus by 7 days post injury (dpi) marked by alcian blue, which is then mostly converted to bone by 14 dpi marked by alizarin red, and subsequently remodeled to the original anatomy by 4–6 weeks, indicating successful regeneration (Fig. 1a).

To characterize the expression of Hh ligands in response to injury we harvested rib tissue from day 0 (uninjured), 3 dpi, and 5 dpi animals (Fig. 1b) and performed fluorescent RNA in situ hybridization (RNA-ISH) for the expression of the three Hh ligands. At these early time points, the resection gap has already been filled in with a mixture of presumed fibroblasts, SSPCs, and other cell types, but chondrogenesis has not yet begun (Fig. 1c), suggesting that prior to 5 dpi is a key window of time for chondrogenic signaling. Similar to previous reports *Dhh, Ihh* and *Shh* are expressed in a few sparse cells at low levels within the bone marrow compartment of the uninjured rib^19–21^ (Fig. 1d) while expression in expected locations such as the testis and growth plate is strong (Supplementary Fig. 1a, b)^22–24^. However expression was not detected in the cortical bone, periosteum, and adjacent muscle. These results suggest that uninjured animals have some baseline expression of *Dhh, Ihh* and *Shh* ligands within the BM of uninjured ribs, whereas there is a minimal baseline Hh expression in the cortical bone, periosteum, and skeletal muscle compartments. At 3 dpi, *Dhh* and *Ihh* are largely undetectable within the resection gap and adjacent tissues, whereas *Shh* is prominently expressed in the central resection gap (Fig. 1d). At 5 dpi, expression of *Ihh* and *Dhh* remain undetectable within the injury site and adjacent tissues, whereas *Shh* expression has expanded and increased in intensity, creating a domain of mesenchymal cells with high *Shh* expression within the central resection gap (~94% of the cells in a region of interest within the central 1/3 of callus are *Shh*+) and adjacent skeletal muscle interstitium (Fig. 1d). In addition, low-expressing cells also can be found near the periphery, consistent with their origin from the surrounding connective tissue (~21% of the cells in a region of interest nearest the 1/3 adjacent to cut ends are *Shh*+) (Supplementary Fig. 1e).

We were surprised to find minimal expression of *Ihh* after injury, since our work previously identified *ihha* activity in large-scale regeneration of zebrafish facial bones^25^. We, therefore, sought to verify the lack of *Ihh* expression in murine rib regeneration by an independent method. To do so, we utilized *Ihh;*LacZ mice, which have a LacZ reporter cassette knocked into the first exon of the *Ihh* gene, allowing us to use LacZ activity as a readout of *Ihh* expression (Supplementary Fig. 1c)^26^. Xgal staining of these animals (Supplementary Fig. 1d) showed expression in the growth plate as expected, but no detectable LacZ expression at 3 dpi. At 7 dpi we did detect LacZ expression in large, maturing chondrocytes as expected. However, since our previous work showed that *Hh* signaling is required prior to the onset of chondrogenesis^7,25^, we determined that this *Ihh* expression in maturing chondrocytes is outside the early window required for stimulation of initial cartilage callus generation. Thus, these data provide an independent line of evidence to support the RNA-ISH data in Fig. 1 demonstrating that *Ihh* is not strongly expressed within the resection site prior to generation of the cartilage callus.

Together these data indicate that *Shh*, but not *Ihh* or *Dhh*, is expressed during the early response to a rib resection injury. Further, the domain of *Shh* expression lies predominantly within the central region of the rib resection site, where a subset of mesenchymal cells begin to upregulate *Shh* expression by 3 dpi and increase expression at 5 dpi. Thus, *Shh* is expressed at the right time and place to activate SSPC chondrogenesis during early callus formation following rib resection injury.

### *Shh* is required for callus generation and subsequent large-scale rib regeneration

**Fig. 2:**
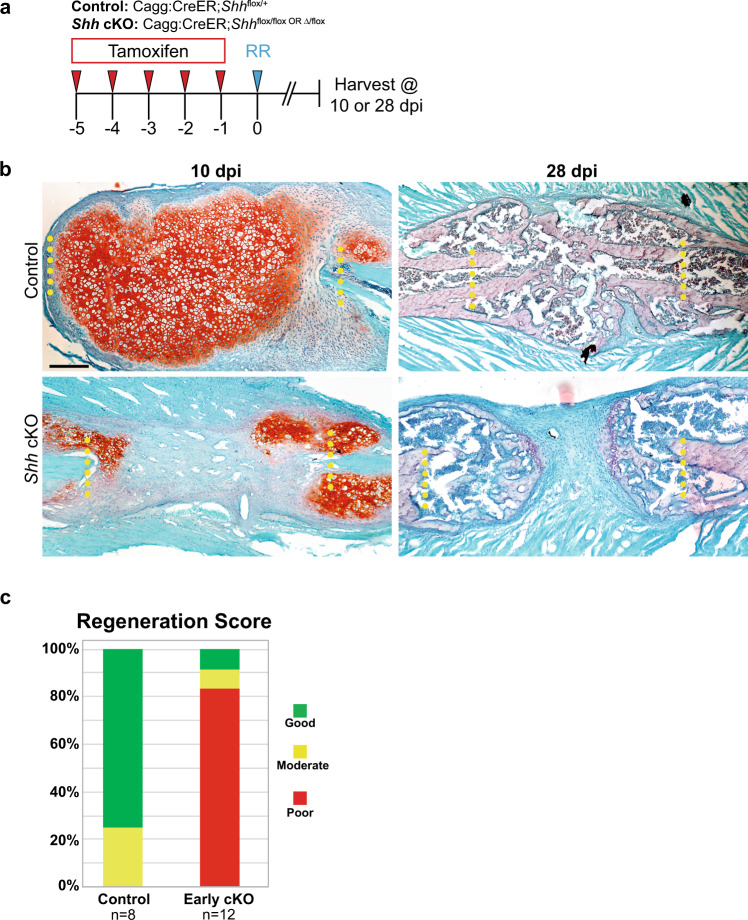
*Shh* is required for callus generation and subsequent large-scale rib regeneration. **a** Experimental overview. To evaluate knock out of *Shh* globally, Cagg:CreER;*Shh*^flox/+^ controls and Cagg:CreER*;Shh*^flox/flox^ or Cagg:CreER*;Shh*^*Δ/*flox^ experimental animals (*Shh* cKO) received tamoxifen injections on days −5, −4, −3, −2, and −1. Resections were performed on day 0 and tissue was harvested at 10 or 28 dpi to assess regeneration outcomes. **b** Safranin-O stained histological sections at 10 and 28 dpi. At 10 dpi, control animals generate calluses mainly composed of cells with chondrocyte morphology, whereas a large callus does not form in *Shh* cKO mice. Only small amounts of Safranin-O stained cartilage can be seen adjacent to the cut sites. At 28 dpi, controls have successfully converted the large cartilage callus to bone that fully spans the defect site. Global *Shh* cKO mice are similarly able to convert cartilaginous regions adjacent to the cut sites to bone, however the central portion of the callus remains undifferentiated and the injury site remains unbridged resulting in a non-union. Scale bar = 200 microns. **c** Blinded scoring of regeneration outcomes. Safranin-O stained sections were used to score regeneration outcomes as Good, Moderate, or Poor (see Methods for details regarding scoring criteria). Overall, controls largely heal well (6/8 animals), whereas *Shh* cKO animals largely heal poorly (10/12 animals). Animals from 10 and 28 dpi were pooled together for graphical representation.

To test if *Shh* if is required for regeneration, we assayed rib repair in CAGG:CreER mice in which we conditionally deleted (cKO) *Shh* globally in the animals prior to injury. To induce global *Shh* cKO in adult animals, we administered tamoxifen daily for five consecutive days prior to rib resection (Fig. 2a). We then assayed callus formation histologically at 10 and 28 dpi to assess cartilage and bone callus production, respectively. At 10 dpi, control mice (CAGG:CreER*;Shh*^flox/+^*)* generate a large, robust cartilage callus that fully spans the defect site, whereas *Shh* cKO mice show cartilage formation near the cut ends but largely fail to generate cartilage in the most central region of the injury site (Fig. 2b). At 28 dpi, control animals generate a large bone callus that fully spans the resection site, whereas the calluses of *Shh* cKO mice remain unbridged indicating a non-union and show only a modest amount of newly generated bone near the cut ends (Fig. 2b). We then used Safranin-O stained sections to blindly score regeneration outcomes as Good, Moderate, or Poor (see Methods for details regarding scoring criteria). Overall, controls largely heal well (6/8 animals), whereas *Shh* cKO animals largely heal poorly (10/12 animals). Thus, *Shh* is required for bridging cartilage callus formation and efficient bone regeneration.

### *Shh* is dispensable for large-scale rib regeneration after 5 dpi

**Fig. 3:**
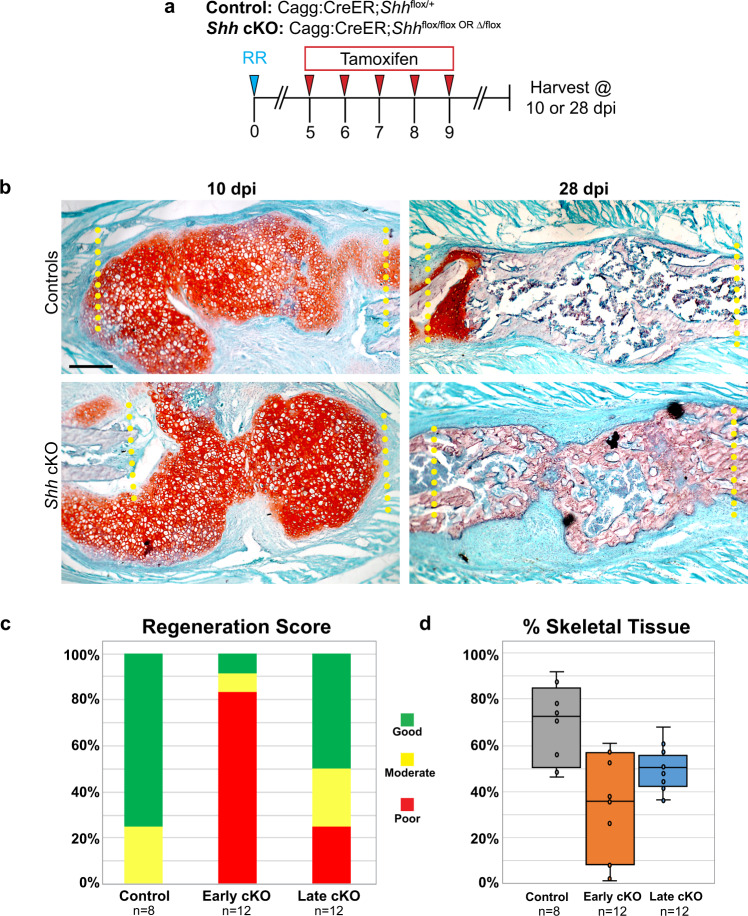
*Shh* is not required for large-scale rib regeneration after 5 dpi. **a** Experimental overview. To evaluate knock out of *Shh* globally, Cagg:CreER;*Shh*^flox/+^ controls and Cagg:CreER*;Shh*^flox/flox^ or Cagg:CreER*;Shh*^*Δ/*flox^ experimental animals received tamoxifen injections on days 5, 6, 7, 8, and 9 post injury. All rib resections were performed on day 0 and rib tissue was harvested at 10 or 28 dpi to assay regeneration outcomes. **b** Controls and experimental mice generate a large Safranin-O stained callus at 10 dpi. By 28 dpi, both genotypes have both successfully converted the cartilage callus to bone that spans the defect site. Scale bar = 200 microns. **c** Blinded scoring of regeneration outcomes. Safranin-O stained sections were used to score each animal’s regeneration outcome as Good, Moderate, or Poor (see Methods for details). Control animals (tamoxifen early and late) largely healed well and scored as Good (6/8 animals) or Moderate (2/8 animals). Early *Shh* cKO animals healed poorly (10/12 animals). Late *Shh* cKO animals mostly healed well and scored as Good (6/12 animals) or Moderate (3/12). Animals were pooled from 7, 10, and 28 dpi time points for each group. **d** Quantification analysis of skeletal tissue (% of resection filled with new skeletal tissue) was performed using samples scored in Fig. 3c. Impaired skeletal regeneration in Early *Shh* cKO animals compared to control animals is evident (−37.3%, 95% CI = [−54.0% to −20.5%]). Late *Shh* cKO largely rescues regeneration compared to early *Shh* cKO animals but does not rescue completely compared to controls (−19%, 95% CI = [−35.9 to −2.96]). See Methods for quantification details.

Our RNA-ISH analysis above (Fig. 1d) demonstrates that *Shh* expression is detectable as early as 3–5 dpi, however this does not rule out an important role for *Shh* during later stages of repair, such as in promoting the proliferation and maturation of chondrocytes^27^. To test if *Shh* is required at the later time points after injury, we genetically deleted *Shh* globally using *Shh* cKO mice. In these experiments, we began 5 days of tamoxifen administration at 5 dpi (Fig. 3a). With this tamoxifen regimen, we aimed to induce a cKO of *Shh* expression after the initial wave described in Fig. 1d. We then assayed callus formation histologically at 10 and 28 dpi to assess cartilage and bone callus production, respectively. At 10 dpi, both control and *Shh* cKO mice generate a large, robust cartilage callus that fully spans the defect site (Fig. 3b). At 28 dpi, both control and *Shh* cKO animals display a bone matrix callus that fully spans the resection site (Fig. 3b). Blinded scoring of regeneration success further corroborated similar regeneration outcomes in control and Late *Shh* cKO animals where the majority of animals were scored as Good and Moderate, in stark contrast to the Early *Shh* cKO animals from Fig. 2 where the majority of animals healed Poorly (Fig. 3c). These observations are further supported by the outcome of quantifying callus skeletal tissue (Fig. 3d). Thus, while Late cKO animals do not heal as robustly as controls, these results show that *Shh* is not required to build a lesion-spanning callus when removed after 5 dpi.

**Fig. 4:**
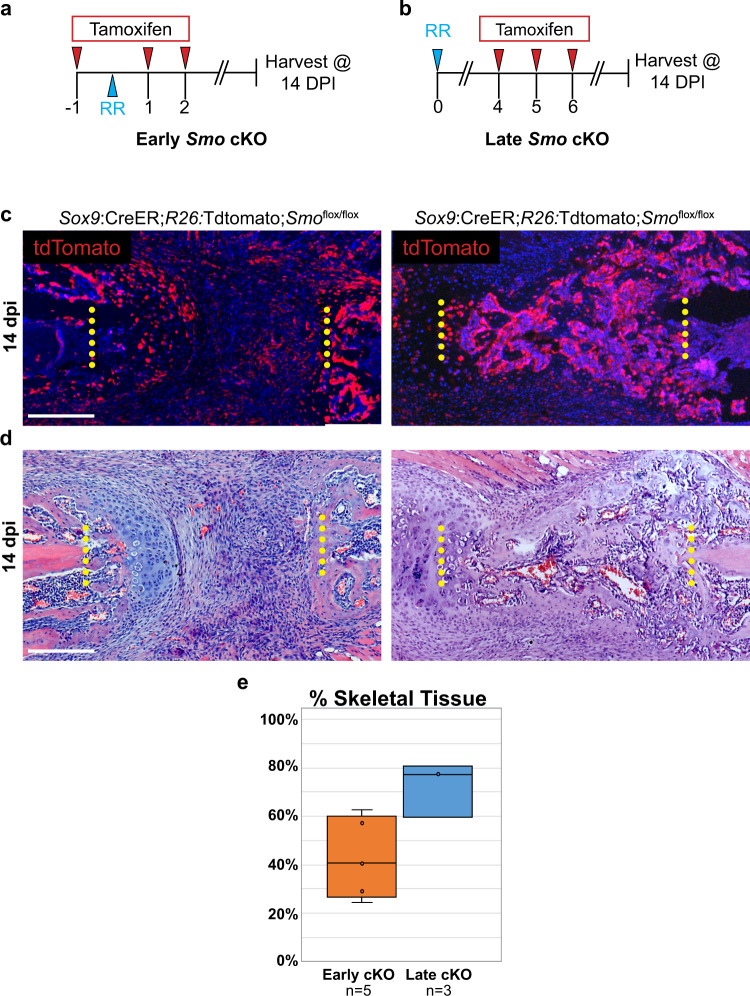
*Smo* is dispensable in callus cells after 4 dpi in a large-scale injury model. **a** To broadly knock out *Smo* in callus cells at the time of injury (“Early *Smo* cKO”), *Sox9:*CreER*;R26:*tdTomato*;Smo*^flox/flox^ animals received tamoxifen injections on −1, 1, and 2 dpi. All rib resections were performed on day 0 and tissue was harvested at 14 dpi to assay regeneration outcomes. **b** To broadly knock out *Smo* in callus cells at 4 dpi (“Late *Smo* cKO”), *Sox9:*CreER*;R26*^tdT^*;Smo*^flox/flox^ animals received tamoxifen injections at 4, 5, and 6 dpi. All rib resections were performed on day 0 and tissue was harvested at 14 dpi to assay regeneration outcomes. **c** tdTomato (tdT) reporter expression shows that *Sox9:*CreER targets the majority of the callus when tamoxifen is administered according to the scheme outlined in A and B. Scale bar = 200 microns. **d** At 14 dpi, H&E staining reveals that Early *Smo* cKO mice generate some bone and cartilage adjacent to the cut ends, but they largely fail to differentiate in the central region of the callus. Late *Smo* cKO mice generate a substantial callus that is largely composed of bone. *N* = at least three for each regimen. Surgical cut sites are indicated with yellow dotted lines. Scale bar = 200 microns. **e** Quantification analysis of H&E sections at 14 dpi reveals impaired skeletal regeneration in early *Smo* cKO animals compared to late *Smo* cKO animals (−29.9%, 95% CI = [−57.2–−2.61]). See Methods for quantification details.

We also tested the timing of requirement for the Hh signaling component, *Smo*, in callus cells using two different tamoxifen regimens. In the *early* KO approach, we treated *Sox9*:CreER;*R26*:tdTomato;*Smo*^flox/flox^ animals with three doses of tamoxifen around the time of injury at −1, 1, and 2 dpi (Fig. 4a), whereas in the *late* KO approach we administered tamoxifen at 4, 5, and 6 dpi (Fig. 4b). Since *Sox9:*CreER is widely upregulated in callus cells in response to local injury^7^, both of these tamoxifen regimens broadly target many cells in the injury milieu, as indicated by the broad expression of the tdTomato lineage trace (Fig. 4c).

To monitor healing progress we harvested tissue at 14 dpi and analyzed H&E-stained histological sections. We found that when *Smo* is removed in the early cKO group, we observed only a modest amount of new skeletal tissue generated near the cut ends, and the central portion of the resection site fails to properly differentiate (Fig. 4d), similar to global *Shh* cKO animals (Fig. 2b). In contrast, in the late *Smo* cKO animals we found that animals build a large bony callus by 14 dpi which fully spans the defect, suggesting a normal healing progression (Fig. 4d). Quantification analysis further supports the observation that early *Smo* cKO animals have reduced skeletal regeneration at 14 dpi compared to late *Smo* cKO animals (Fig. 4e).

Taken together, these results support the existence of a critical window for Hedgehog activity during the first 5 days of large-scale rib regeneration to generate a lesion-spanning callus and that loss of *Shh* or *Smo* after this window does not significantly affect early callus formation.

### *Shh* expression outside of Sox9+ SSPCs

**Fig. 5:**
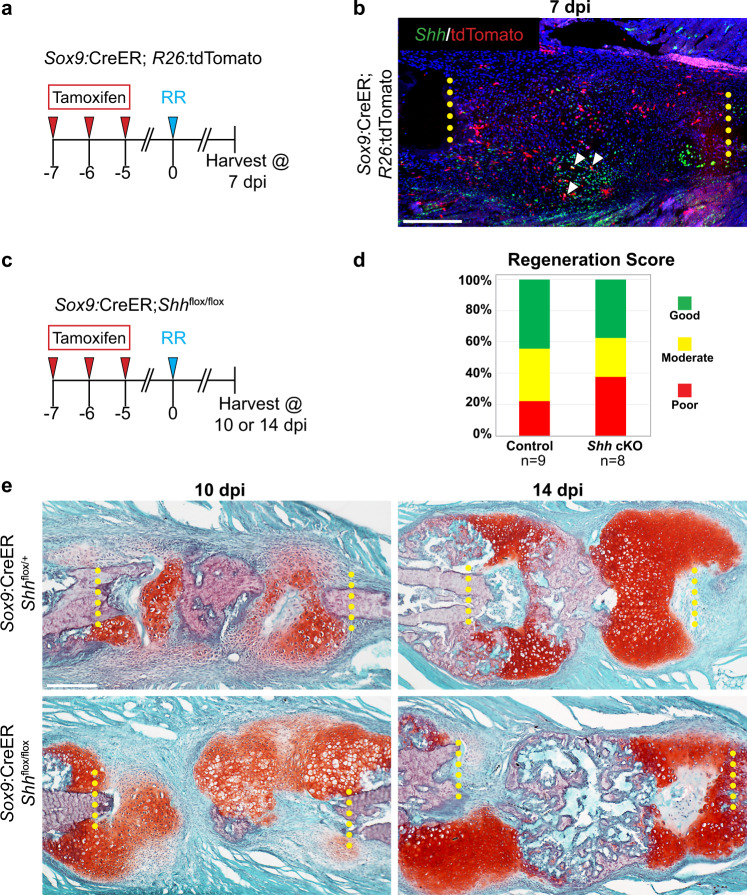
The primary source of SHH is not *Sox9*-derived SSPCs. **a** Experimental overview. To lineage-trace *Sox9*+ lineage cells, *Sox9:*CreER*;R26:*tdTomato animals received tamoxifen injections on days −7, −6, and −5. All rib resections were performed on day 0 and tissue was harvested at 7 dpi to assay *Shh* expression in **b**. **b** RNA-ISH for *Shh* (green) on histological sections at 7 dpi. Sox9+ lineage cells express tdTomato (tdT, red). *Shh* expression is strongly detected within cells located in the central resection gap and in the surrounding periosteal tissues. Although a few tdT+ lineage-traced cells express *Shh* (white arrowheads), the majority of *Shh*-expressing cells are tdT-negative. Surgical cut sites are indicated with yellow dotted lines. *N* = 3. Scale bar = 200 microns. **c** Experimental overview. To knock out *Shh* in Sox9+ lineage cells, *Sox9:*CreER*;Shh*^flox/flox^ animals received tamoxifen injections on days −7, −6, and −5. All rib resections were performed on day 0 and tissue was harvested at 10 or 14 dpi to assay regeneration outcomes. **d** Blinded scoring of regeneration outcomes. Histological sections were used to score regeneration outcomes as Good, Moderate, or Poor (see Methods for details regarding scoring criteria). Control animals largely healed well and scored as Good (4/9 animals) or Moderate (3/9 animals). Similarly, *Sox9:*CreER*;Shh*^flox/flox^ animals largely healed well (3/8 animals) or Moderate (2/8). Animals from 10 and 14 dpi were pooled for graphical representation. **e** At 10 dpi, *Sox9:*CreER*;Shh*^flox/+^ controls and *Sox9:*CreER*;Shh*^flox/flox^ mice both generate a large Safranin-O stained callus composed largely of cells with chondrocyte morphology and with the central-most portion beginning osteogenic differentiation. At 14 dpi, *Sox9:*CreER*;Shh*^flox/+^ controls and *Sox9:*CreER*;Shh*^flox/flox^ mice have large Safranin-O-stained calluses with a central region undergoing bone conversion. Surgical cut sites are indicated with yellow dotted lines. Scale bar = 200 microns.

Our previous work identified a population of periosteal resident SSPCs that can be marked with a *Sox9*:CreER transgene, and that requires the Hedgehog signaling component *Smo* for proper large-scale rib regeneration^7^. We next asked if Sox9+ lineage SSPCs are a cellular source of SHH, which in combination with our previous results, would reflect an autocrine SHH*-Smo* signaling mechanism. To test this hypothesis, we performed RNA-ISH for *Shh* on sections harvested from *Sox9*:CreER*;R26:*tdTomato double transgenic animals. To induce lineage-tracing of Sox9+ SSPCs, we administered tamoxifen to *Sox9*:CreER*;R26:*tdTomato animals for three consecutive days beginning 7 days prior to surgery and harvested rib tissue at 7 dpi (Fig. 5a). At this stage, most *Shh*-expressing cells (green) were tdTomato (tdT) negative (~96%) (Fig. 5b). We were, on occasion, able to detect a few tdT+ cells that were also expressing *Shh*, although almost all tdT+ cells were *Shh* negative (~94%). Thus, *Shh* is primarily expressed in tdT-negative cells, suggesting that the primary source of SHH is not from the Sox9+ SSPC lineage.

To confirm that Sox9+ lineage SSPCs are not a critical source of SHH protein, we conditionally deleted *Shh* in *Sox9*-expressing cells using *Sox9*:CreER*;Shh*^flox/flox^ animals prior to injury and assayed rib regeneration. To induce *Shh* cKO in the Sox9+ lineage, we administered tamoxifen to *Sox9*:CreER*;Shh*^flox/flox^ animals for three consecutive days beginning 7 days prior to surgery and harvested rib tissue at 7 and 14 dpi (Fig. 5c). In both controls and *Shh* cKO animals, we observed large Safranin-O positive cartilage calluses at 7 dpi and cartilage calluses with significant regions converted to bone at 14 dpi (Fig. 5e). Blinded scoring of regeneration further corroborated a similar healing capacity of experimental animals compared to controls (Fig. 5d). These data reveal that cKO of *Shh* in *Sox9*-lineage SSPCs has no major effect on large-scale rib regeneration, consistent with the observation that the majority of *Shh* expression occurs outside of the Sox9+ lineage. Thus, Sox9+ lineage cells are not the major source of SHH protein.

### *Shh* is expressed by *Prrx1*-expressing cells and is not dependent upon the presence of Sox9+ lineage cells

**Fig. 6:**
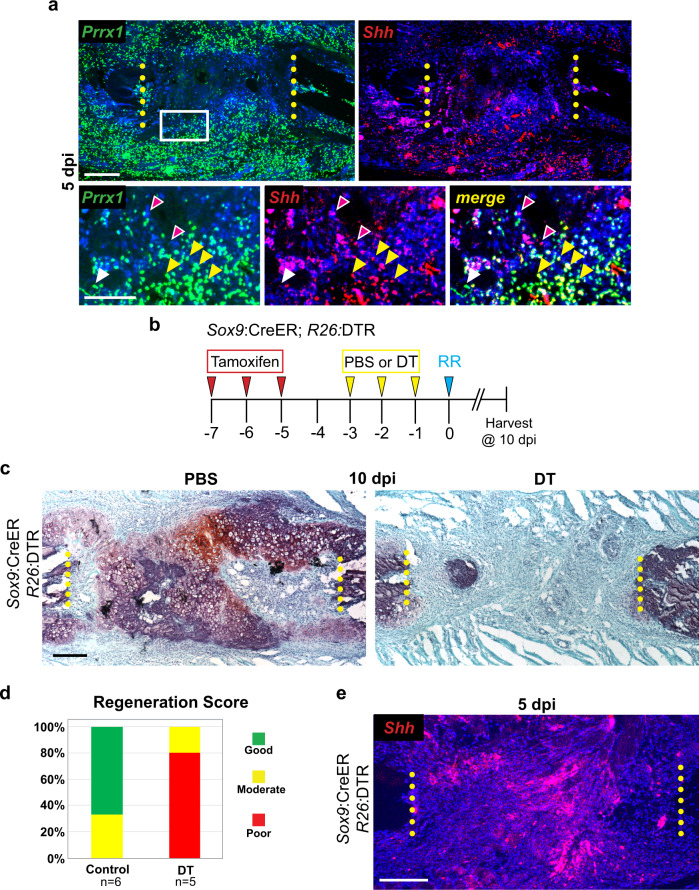
*Shh* is expressed by *Prrx1*-expressing cells and is not dependent upon the presence of Sox9+ lineage cells. **a** RNA-ISH for *Prrx1* (green) at 5 dpi shows a wide distribution of cells throughout the callus. At higher magnification Double RNA-ISH shows that *Shh* positive cells (red) co-express *Prrx1* (yellow arrowheads). Some *Prrx1*-expressing cells are *Shh* negative and vice versa (white and magenta arrowheads respectively). Surgical cut sites are indicated with yellow dotted lines. *N* = 2. Scale bars = 200 microns. **b** Experimental overview of Sox9+ cell ablation regimen. All animals received tamoxifen injections on days −7, −6, and −5. Controls included *Sox9:*CreER*;R26:*DTR animals that received PBS injections on days −3, −2, and −1 and Cre negative *R26:*DTR animals that received DT injections on days −3, −2, and −1. *Sox9:*CreER*;R26:*DTR experimental animals received DT treatment on days −3, −2, and −1. All rib resections were performed on day 0 and rib tissue was harvested at 10 dpi to assess healing progress in **c**. **c** At 10 dpi, *Sox9:*CreER*;R26:*DTR control animals treated with PBS produce a large Safranin-O stained cartilage callus. However, *Sox9:*CreER*;R26:*DTR animals treated with DT fail to build a bridging callus. Surgical cut sites are indicated with yellow dotted lines. Scale bar = 200 microns. **d** Blinded scoring of regeneration outcomes. Safranin-O stained sections were used to score each animal’s regeneration outcome as Good, Moderate, or Poor (see Methods for details). Control animals (early and late tamoxifen) largely healed well and scored as Good (4/6 animals) or Moderate (2/6 animals) whereas *Sox9*-ablation animals largely healed poorly (4/5 animals). All animals scored are 10 dpi. **e** To determine if the ablation of Sox9+ cells impacts the expression of *Shh*, RNA-ISH was performed for *Shh* expression at 5 dpi. *Shh* was still strongly detected within cells located in the central region of the resection and in the surrounding periosteal and skeletal muscle tissues. *N* = 3. Scale bar = 200 microns.

To further characterize *Shh*-expressing cells, we next asked if *Shh* is expressed by mesenchymal cells vs. other cell types present after injury. Since *Prrx1* expression broadly marks mesenchymal cells following injury^28,29^, we performed double RNA-ISH for *Shh* and *Prrx1* at 5 dpi and observed cells with substantial overlap in expression (Fig. 6a). Indeed, nearly all *Shh*-expressing cells (red) are yellow due to co-expression of *Prrx1* (green) (Fig. 6a, yellow arrowheads). In contrast, some *Prrx1*-expresssing cells (green) are *Shh* negative (white arrowheads). We can also on occasion, detect *Shh*+ *Prrx1*− expressing cells (magenta arrowheads), though they are quite rare. Together these data indicate that at 5 dpi, cells expressing *Shh* are largely constrained to a subset of *Prrx1*-expressing mesenchymal cells.

Although *Shh* does is only expressed in small subset of cells in the Sox9+ lineage SSPCs (Fig. 5b), we wondered if the expression of *Shh* in *Prrx1*-expressing cells is dependent upon the presence of Sox9+ lineage cells. We, therefore assayed *Shh* expression in animals in which Sox9+ lineage cells were selectively ablated with diphtheria toxin (DT) prior to injury^30^. To selectively ablate *Sox9*+ cells prior to injury, we generated *Sox9*:CreER;*R26*:DTR double transgenic mice. In this system, tamoxifen administration induces expression of the diphtheria toxin receptor (DTR) specifically in Sox9+ cells, rendering Sox9+ cells susceptible to subsequent diphtheria toxin (DT) exposure. To ablate Sox9+ cells prior to injury, we administered three daily injections of tamoxifen to *Sox9*:CreER;*R26*:DTR mice starting 7 days before injury. Mice were then treated with PBS (Control) or Diphtheria Toxin (DT) daily for 3 days prior to injury (Fig. 6b). Similar to our previous results where *Smo* was removed from Sox9+ lineage cells^7^, DT-treated *Sox9*:CreER;*R26*:DTR animals fail to generate a cartilage callus at 10 dpi, whereas PBS-treated animals generate large cartilage calluses (Fig. 6c). Blinded scoring of regeneration success at 10 dpi further corroborated impaired regeneration of DT-treated *Sox9*:CreER;*R26*:DTR animals vs. PBS-treated controls (Fig. 6d). To determine if *Shh* is expressed even in the absence of these Sox9+ SSPCs, we harvested tissue from DT-treated *Sox9*:CreER;*R26*:DTR mice at 5 dpi and performed RNA-ISH. At 5 dpi, *Sox9*:CreER;*R26*:DTR animals still broadly express *Shh* within the injury site (Fig. 6e) similar to wild-type animals (Fig. 1), supporting the idea that the expression of *Shh* in the local injury environment by *Prrx1-*expressing cells is independent of the presence of Sox9+ lineage SSPCs.

Together, these data provide multiple independent lines of evidence to support a model in which *Shh* is broadly upregulated in the local injury environment by Prrx1-expressing cells following large-scale rib injury and its expression is independent of Sox9+ lineage cells. Thus in large-scale rib repair, we conclude that *Shh* is required in a predominantly *Prrx1-expressing; Sox9-*negative mesenchymal population.

### Hedgehog signaling is largely dispensable for small-scale bone repair

**Fig. 7:**
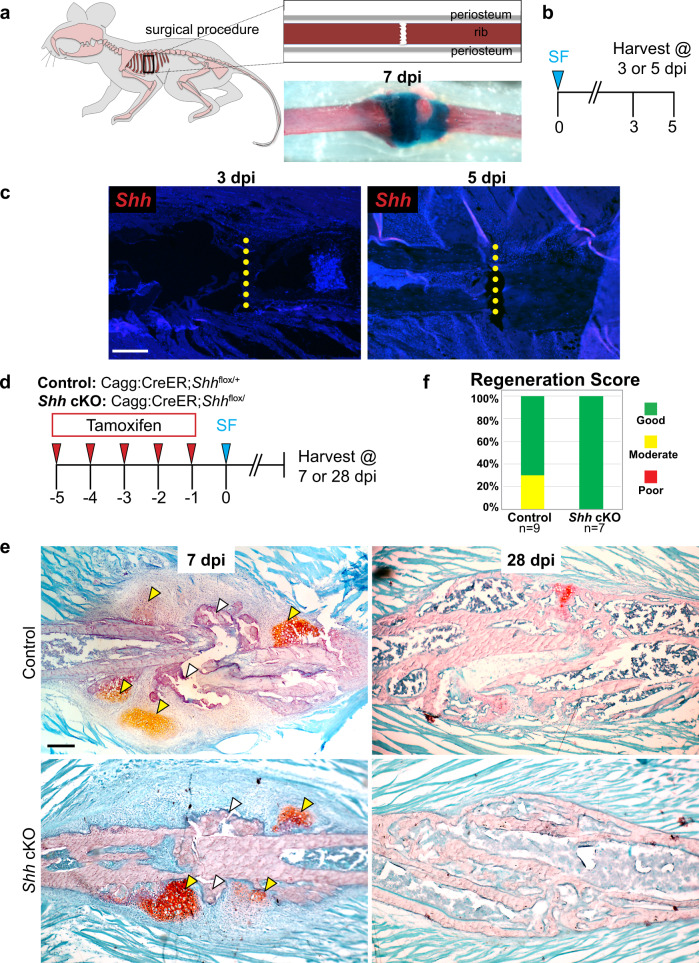
*Shh* is not required for small-scale repair. **a** Overview of surgical fracture procedure. Briefly, this injury model includes creating a surgical fracture of the rib bone. At 7 dpi, bridging of the injury site can be observed with an alcian blue stained callus. **b** Surgical fractures were performed at day 0 and tissues were harvested at 3 or 5 dpi for analysis in **c**. **c** RNA-ISH for *Shh* on histological sections of tissue harvested at 3 dpi or 5 dpi shows that *Shh* is largely undetectable at both 3 and 5 dpi. Scale bar = 200 microns. *N* = 2. **d** To evaluate knock out *Shh* globally, Cagg:CreER;*Shh*^flox/+^ controls and Cagg:CreER*;Shh*^flox/flox^ or Cagg:CreER*;Shh*^*Δ/*flox^ experimental animals received tamoxifen injections on days −5, −4, −3, −2, and −1. All surgical fractures were performed on day 0 and tissue was harvested at 7 or 28 dpi to assay regeneration outcomes. **e** At 7 dpi, controls and *Shh* cKO experimental animals generate a Safranin-O stained callus with cartilage morphology. Yellow arrowheads indicate areas where cartilage is forming while white arrowheads indicate forming bone. At 28 dpi, both controls and *Shh* cKO animals have successfully converted the cartilage callus to bone that fully spans the injury site. Scale bar = 200 microns. **f** Blinded scoring of regeneration outcomes. Safranin-O stained sections were used to score each animal’s regeneration outcome as Good, Moderate, or Poor (see Methods for details). Control animals largely healed well and scored as Good (6/9 animals) or Moderate (3/9 animals). *Shh* cKO animals also healed well and all scored as good (7/7 animals). Animals were pooled from 7 and 28 dpi time points for each group.

During the above experiments, we noticed that (1) cells expressing *Shh* are predominantly found in the most central portion of the resection gap, and (2) that *Shh* and *Smo* cKO mice fail to regenerate this central region, yet still generate callus tissue adjacent to the edges of the cut rib ends (*Hh*-independent repair). We, therefore, hypothesized that *Shh* is uniquely required for regeneration in large-scale contexts in which *Shh*-independent callus production at the cut ends is insufficient to bridge the injury gap. To test this hypothesis, we created a small-scale surgical fracture in the rib bone, leaving adjacent periosteal and muscle tissues intact (Fig. 7a). In this model, a small alcian blue stained cartilage callus forms by 7 dpi, indicating an endochondral repair pathway (Fig. 7a); bridging is complete by ~28 dpi.

We first asked if *Shh* is expressed in this small-scale injury context. To assay *Shh* expression, we performed RNA-ISH for *Shh* at 3 and 5 dpi following surgical fracture. At both time points, *Shh* expression was not detectable (Fig. 7c). These data suggest that in contrast to a large-scale injury context, *Shh* is not upregulated during early callus formation in the context of small-scale bone injury.

To further determine if *Shh* is a critical mediator of small-scale bone repair, we conditionally deleted *Shh* in *Shh* cKO mice prior to surgical fracture and then assayed repair. To induce global cKO of *Shh* in adult animals, we administered tamoxifen daily for 5 days prior to injury (Fig. 7d). We then assayed callus formation histologically at 7 and 28 dpi to assess cartilage and bone callus production, respectively (Fig. 7e). At 7 dpi, both CAGG:CreER*;Shh*^flox/+^ controls and *Shh* cKO mice generated small, but notable cartilage nodules within the periosteum adjacent to the injury site. We also observed that the cut ends of both controls and *Shh* cKO animals abut each other and are bridged by newly generated irregular skeletal tissue. At 28 dpi, both control and *Shh* cKO animals generate a bone callus that fully spans the injury site, indicating successful healing in both genotypes (Fig. 7e). Blinded scoring of regeneration outcomes further corroborates similar healing progression in control and *Shh* cKO animals (Fig. 7f). Thus, *Shh* is largely dispensable for small-scale rib repair.

**Fig. 8:**
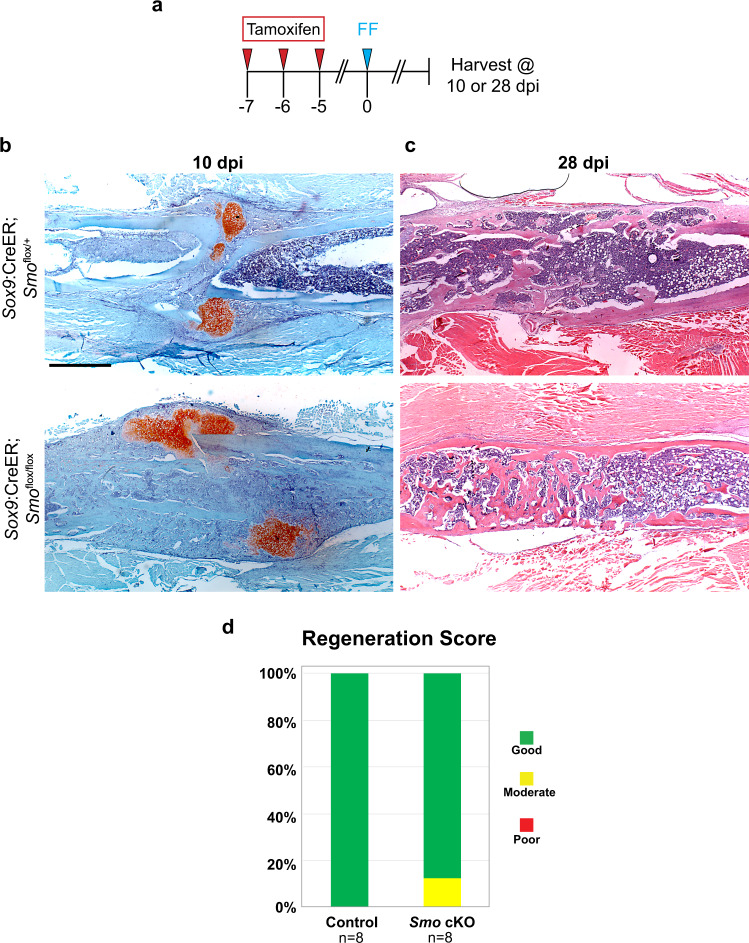
*Smo* is not required in Sox9+ lineage cells for femur fracture repair. **a** To knock out *Smo* in Sox9+ lineage cells, animals received tamoxifen injections on days −7, −6, and −5. All femur fractures were performed on day 0 and tissue was harvested at 10 or 28 dpi to assay regeneration outcomes. **b** Safranin-O stained histological sections at 10 dpi show that both *Sox9*:CreER;*Smo*^*flox/+*^ controls and *Sox9*:CreER;*Smo*^*flox/flox*^ *Smo* cKO mice generate a Safranin-O stained cartilage callus. *N* = 4. Scale bar = 1 mm. **c** H&E stained histological sections at 28 dpi. Both *Sox9*:CreER;*Smo*^*flox/+*^ controls and *Sox9*:CreER;*Smo*^*flox/flox*^ *Smo* cKO mice exhibit an injury sited bridged with bone. *N* = 4. **d** Blinded scoring of regeneration outcomes. Safranin-O (10 dpi) and H&E (28 dpi) stained sections were both used to score each animal’s regeneration outcome as Good, Moderate, or Poor (see Methods for details). Control (*Sox9*:CreER;*Smo*^flox/+^) animals all healed well and scored as Good (8/8 animals). *Smo* cKO (*Sox9*:CreER*;Smo*^flox/flox^) animals also healed well and scored as Good (7/8 animals) or Moderate (1/8 animals).

We next set out to determine if Hh signaling plays a major role in other small-scale repair contexts. Global cKO of *Smo* and widespread pharmacological inhibition of Hh signaling during fracture repair was previously investigated by two other groups^8,31^. A mild-moderate compromise in callus formation is reported in both studies, however mice ultimately form complete unions. As the requirement for *Shh* in Sox9+ cells couldn’t be specifically evaluated in these prior experiments, here we specifically tested if *Smo* is required in the Sox9+ lineage cells during femur fracture repair using the pre-injury tamoxifen regimen. To induce *Smo* KO in Sox9+ lineage SSPCs, we administered tamoxifen to *Sox9*:CreER*;Smo*^*flox/flox*^ animals for three consecutive days beginning 7 days prior to injury (Fig. 8a). We then induced an impact femur fracture at day 0 and harvested femur tissue at 10 and 28 dpi to evaluate cartilage and bone production, respectively. At 10 dpi in both *Sox9*:CreER*;Smo*^*flox/*+^ controls and *Sox9*:CreER*;Smo*^*flox/flox*^ animals, a large Safranin-O positive cartilage callus of similar size is evident (Fig. 8b). At 28 dpi, both controls and *Smo* KO animals have a large bony callus that completely spans the injury site (Fig. 8c). Blinded scoring of regeneration outcomes further corroborates similar healing progression in control and *Shh* cKO animals (Fig. 8d). These results suggest that Hh signaling in Sox9+ SSPCs is not required for full-union fracture repair.

### Single-cell RNA sequencing reveals a reduction in *Cxcl12-*expressing cells when Sox9+ lineage cells do not receive a Hh signal

**Fig. 9:**
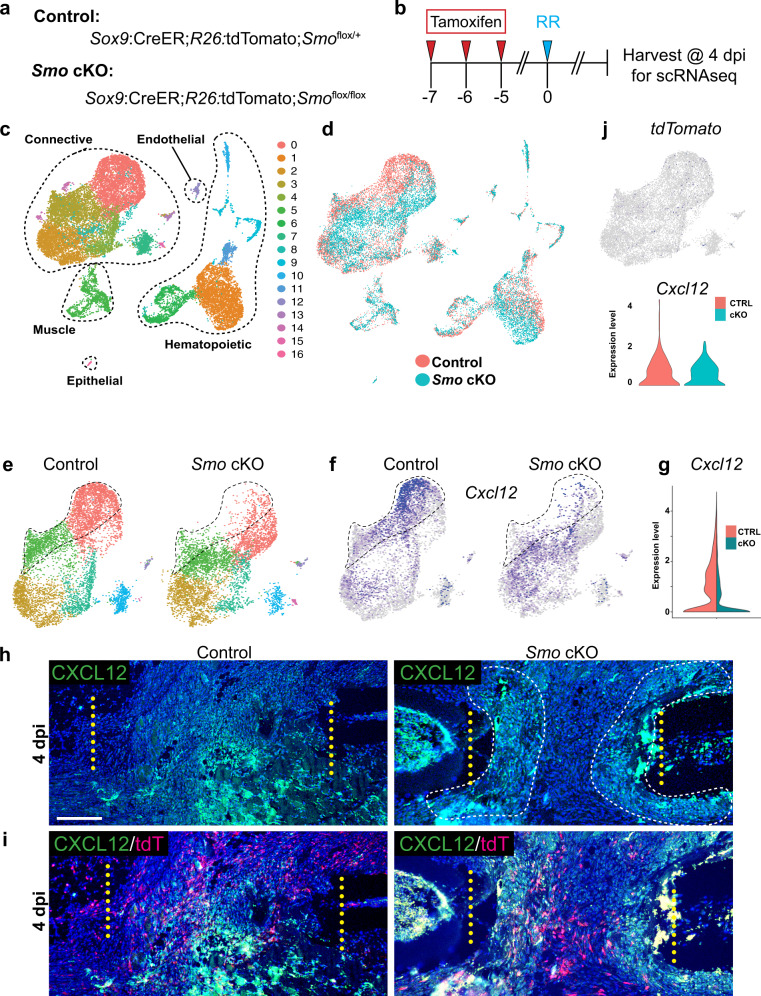
Activation of Sox9+ lineage cells by Hh signaling is required for *Cxcl12* expressing cells to populate the early callus. **a** Mice used for single-cell RNA sequencing (scRNAseq) included *Sox9:*CreER*;Smo*^*flox/+*^ (controls) and *Sox9*:CreER;*Smo*^flox/flox^(*Smo* cKO) animals. **b** To knock out *Smo* in *Sox9*+ lineage cells, animals received tamoxifen injections on days −7, −6, and −5. Rib resections were performed at day 0 and callus tissue was harvested for scRNAseq at 4 dpi. **c** UMAP visualization identified 17 transcriptional cell states broadly representing the major cell types expected, including connective, endothelial, hematopoietic, and muscle cells. **d**, **e** UMAP visualization reveals a shift in connective tissue cluster 0 cells between control and *Smo* cKO animals. **f** Feature plot identifies *Cxcl12* as marker of cluster 0 that is depleted in *Smo* cKO compared to control animals. **g** Violin plot of control vs. *Smo* cKO animals reveals decreased expression of *Cxcl12* in cluster 0 of *Smo* cKO animals. **h** An IF assay for CXCL12 in control vs. *Smo* cKO animals reveals a reduction of cells expressing CXCL12 in *Smo* cKO animals and their spatial restriction to the region adjacent to the cut ends (white dotted lines). *N* = 2 for each genotype. Scale bar = 200 microns. **i** Immunofluorescence assay for CXCL12 expression combined with imaging for the Tdtomato lineage trace of Sox9+ cells in control vs. *Smo* cKO animals reveals that CXCL12-expressing and Sox9+ lineage cell populations appear quite distinct, as minimal overlap between the tdTomato and CXCL12 signal is observed. *N* = 2 for each genotype. **j** Feature plot shows that cells expressing *tdTomato* do not strongly correlate with those expressing high levels of *Cxcl12*, suggesting these populations are distinct. A violin plot of sub-setted tdTomato+ cells shows similar *Cxcl12* expression levels between control and *Smo* cKO animals.

The experiments above, as well as our previous work, demonstrate that activation of the Hh pathway in periosteal cells is required for proper cartilage callus formation, yet how Hh activity in periosteal SSPCs ultimately orchestrates repair is unknown. To investigate this question, we used single-cell RNA sequencing (scRNAseq) to characterize the transcriptional landscape of the early repair callus in controls (*Sox9*:CreER*;R26:*tdTomato;*Smo*^*flox/*+^*)* vs. Sox9+ lineage *Smo* knockout (cKO) animals (*Sox9*:CreER*;R26:*tdTomato*;Smo*^*flox/flox*^*)* (Fig. 9a). To induce *Smo* cKO, we administered tamoxifen to *Sox9*:CreER*;R26:*tdTomato*;Smo*^*flox/flox*^ for three consecutive days beginning 7 days prior to surgery and collected fresh, dissociated callus cells at 4 dpi, and performed scRNAseq using the 10x Genomics Chromium platform (Fig. 9b). We chose 4 dpi as it is within the critical window for Hh activation. UMAP visualization identified ~17 transcriptional cell states broadly representing the major cell types expected, including connective, endothelial, hematopoietic, and muscle cells (Fig. 9c, Supplementary Fig 2).

To assess the activity of SSPCs, we specifically compared the cluster of connective-type cells from control vs. *Smo* cKO animals and revealed a dramatic shift in Cluster 0 cells upon *Smo* deletion (Fig. 9d, e). As cluster areas largely overlapped between control and *Smo* cKO contexts, the shift in the Cluster 0 grouping is likely biological and not a technical batch effect (Fig. 9d). We next investigated the genes that best distinguish control vs. *Smo* cKO Cluster 0 cells and determined that *Cxcl12* is expressed highly in large number of Cluster 0 cells in the control context, but the number of cells expressing *Cxcl12* within this cluster is severely reduced in the *Smo* cKO context (Fig. 9f, g). High *Cxcl12* expression has been observed in bone-marrow stromal cells that can adopt an SSPC-like state in response to injury and subsequently be recruited to generate chondrogenic and osteogenic cells of the repair callus^13^. *Cxcl12*-high expressing cells are notably lacking in *Smo c*KO mice, suggesting that *Smo* activity in *Sox9*+ periosteal SSPCs may be required to recruit these *Cxcl12*-expressing bone marrow cells into the repair callus.

To evaluate the expression of *Cxcl12* within control and *Smo* cKO contexts, we performed an immunofluorescence assay for CXCL12 at 4 dpi in control vs. *Smo* cKO animals. In control animals, we observe a broad distribution of *Cxcl12-*expressing cells throughout the callus, with the highest expressing cells located in the most central region of the repair callus (Fig. 9h). In contrast, in *Smo* cKO animals, *Cxcl12* expression was primarily localized to an area adjacent to the cut rib bones with little to no expression in the most central region of the repair callus (Fig. 9h). Together, these data support a model of large-scale rib regeneration where *Shh* activation of periosteal Sox9+ SSPCs through *Smo* ultimately leads to recruitment of *Cxcl12*-expressing cells into the most central portion of the early repair callus to facilitate large-scale regeneration.

A similar but alternative interpretation of our scRNAseq data could be that Hh activation of Sox9+ lineage cells stimulates their differentiation into a *Cxcl12*-high expressing population, as described during bone development^32^. However, in our experiments *Cxcl12*-expressing and Sox9+ lineage cell populations appear quite distinct, as we observed minimal overlap between tdTomato fluorescence and CXCL12 protein expression (Fig. 9i). In addition, a UMAP for tdTomato expression reveals little correlation between where these cells cluster and the cluster of high *Cxcl12*-expressing cells that is missing in the mutant (Fig. 9j). Furthermore, a sub-selection for tdTomato-expressing cells from our scRNAseq data did not express markedly different levels of *Cxcl12* in control and *Smo* cKO conditions (Fig. 9j). Thus, these data support a model where *Cxcl12*-high expressing cells are largely a distinct population from the Sox9+ lineage cells and whose presence during repair is dependent on Hh signaling.

## Discussion

Repairing a small vs. large injury may not only necessitate distinct bone healing pathways (direct and/or endochondral) but may also require distinct triggers to initiate these pathways. Our studies provide evidence for *Shh* as a required trigger to initiate a type of repair that creates a bridging callus in the context of a large-scale injury. We were surprised to find *Shh* to be required instead of *Ihh*, as SHH has been traditionally considered a ligand important for embryonic skeletal patterning and discovering a role for SHH in adult skeletal biology was unexpected^33–35^. In addition, it is IHH that has a long-appreciated role in endochondral bone growth^27,36^, and furthermore, our previous work showed that *ihha* rather than *shh* is critical for large-scale regeneration in the zebrafish jaw bone^25^. However, in the context of the mouse rib, *Shh* but not *Ihh* or *Dhh*, is rapidly and broadly upregulated in the first few days following large-scale injury (Fig. 1). Furthermore, genetic deletion of *Shh* prior to injury but not afterward, dramatically impairs regeneration (Figs. 2, 3). It is possible that in mice, *Shh* may be playing a similar role to *ihha* in fish—indicating that the particular type of Hh protein may not matter as much as when and where it is expressed in these divergent species.

So as to prevent the accidental formation of ectopic cartilage or bone, the regulation of these Hh ligand genes in response to injury is likely very tightly controlled, perhaps requiring multiple inputs and/or chromatin alterations. How *Shh* in the mouse and *ihha* in the fish is regulated or how *Shh* is regulated in the murine rib vs. the femur will be a fruitful avenue of future investigation. One approach may be to define regulatory elements that govern *Shh* expression specifically in response to injury and determine if these elements are differentially regulated in femur vs. rib or large-scale vs. small-scale injury. In addition, since *Shh* is an important patterning factor during both limb and rib development^33–35,37^, it is tempting to consider that large-scale regeneration requires the redeployment of a developmental program involving *Shh*, whereas small-scale injuries heal through an independent program of repair that does not mirror developmental history. Whether or not *Shh* expression in large-scale regeneration represents a bona fide reactivation of a developmental program is still to be determined.

**Fig. 10:**
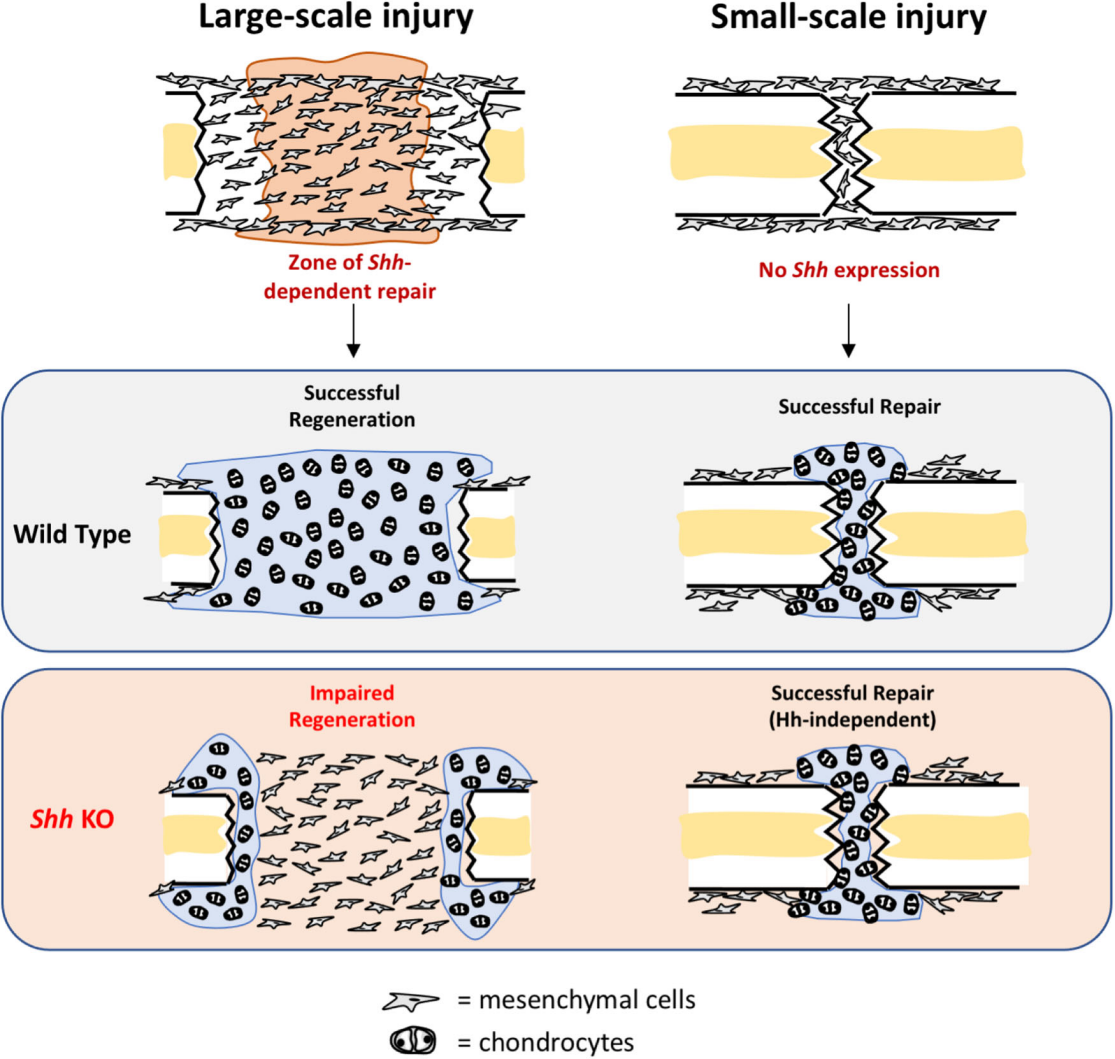
Summary model. In response to large-scale injury in the rib, *Shh* is rapidly and broadly upregulated in *Prrx1*-expressing mesenchymal cells that can be found in the central injury region at 3–5 days post injury (dpi), whereas *Shh* expression is not detectable in response to small-scale injury. In animals where *Shh* is conditionally removed (*Shh* KO), large-scale injuries do not generate cartilage in the central callus area and subsequently do not heal successfully. In contrast, the full repair of small-scale injuries (both in rib and femur) largely does not require Hh signaling. Thus, bone repair is a process that can involve region-specific signaling requirements depending on the injury type and *Shh* is selectively required to build a centrally located bridging callus during large-scale repair.

Our studies reveal that not only is *Shh* required to initiate large-scale rib repair but may be dedicated to building new skeletal tissue within a specific region of the repair callus. Our observation that *Shh* cKO mice still produce some cartilage adjacent to the cut ends and later converts that cartilage to bone supports a model where the regions near the cut ends repair via endochondral ossification employing a Hh-independent mechanism, whereas repair in the central region of the defect site with a bridging callus, also via endochondral ossification, is indeed *Shh*-dependent. Notably, *Shh* cKO animals fail to form a cartilage callus in the most central region of the injury site, while still producing cartilage immediately adjacent to the cut ends. The undifferentiated central portion of the injury site in *Shh* cKO animals mirrors the domain of *Shh* expression in control animals at 3–5 days post injury, further supporting a link between *Shh* expression in the injury environment and subsequent callus differentiation. Together, these observations support a model of large-scale bone regeneration where repair close to the cut bone ends is *Shh-*independent, whereas repair in the most central region of the injury site is critically dependent on early expression of *Shh* (Fig. 10). Why there would be region-specific requirements for *Shh* remains unclear. Perhaps the cells that occupy different regions of the callus are derived from alternative lineages (i.e., periosteal enriched at the cut ends vs. bone marrow enriched in the central region) and therefore may have different required inputs for successful differentiation. It is also worth noting that the injury environment is not uniform and there may be certain environmental conditions (i.e., vascularization, biomechanics) that create “zones” of the callus with different signaling requirements^38–40^.

Intriguingly, a very similar regional requirement for Hh signaling has been observed in regenerating lizard tails. When Hh-inhibiting drugs are applied to lizard injury site after tail amputation, a new long-tail fails to regenerate, yet the terminal vertebra that was injured during amputation successfully repairs through an endochondral pathway that resembles fracture healing^41^. Thus, lizards also exhibit a paradigm where bone healing at the site of injury repairs in a Hh-independent manner, whereas the large-scale regenerative events are indeed Hh-dependent.

The observation that cells located in the region immediately adjacent to the cut bone ends do not generally express high levels of *Shh* has important implications for how SSPC behavior is regulated in response to injury. The most obvious is that a small-scale injury such as a simple fracture does not contain a region of repair tissue distant from the injured bone ends and thus may not express *Shh* at all. Indeed, we are unable to detect *Shh* expression within the first 5 day window following surgical fracture, and global *Shh* cKO in these animals still resulted in complete healing. In addition, loss of *Smo* in the Sox9+ lineage does not prevent small-scale femur fracture repair. These results are consistent with recent work by two groups which revealed that global *Smo* KO and widespread pharmacological inhibition of Hh signaling during fracture repair did not prevent the ultimate union of small-scale injuries^8,31^. One prominent outstanding mystery is which factors or conditions result in *Shh* being expressed following large-scale, but not during small-scale injury. For example, a larger-scale injury may result in more severe hypoxia^42–44^, increased immune response^45^, or an altered mechanical environment which in turn induces *Shh* expression^46–49^. Indeed, biomechanical stability is known to have a major impact on the lineage progression of SSPCs during healing and rigidly stabilize fractures can heal via direct ossification without first building a cartilage callus^50^.

When combined with our observations, these data provide similar lines of evidence in several species and models of skeletal regeneration whereby a Hh ligand initiates repair in a large-scale injury and has a regional role during building a bridging callus. These observations point to a Hh ligand as the critical factor underlying the regeneration of large-scale, but not small-scale injuries. Large- vs. small-scale repair may require different pathways, mechanisms, and regulatory controls. How conserved these paradigms may be in other bones and across other species is yet to be determined.

While our previous work has shown that early Hh signaling is required in Sox9+ lineage SSPCs to build a substantial bridging callus, other cell types may also be responding to Hh signaling during repair. Indeed, our own studies have shown that the receptor *Ptch1* and signaling component *Smo* are also expressed outside the Sox9+ lineage^7^. For example, Hh signaling may have additional roles on endothelial cells to stimulate angiogenesis^51,52^ or may act on cells that are part of the innate or adaptive immune system^53,54^. Whether the activity of SHH on cell types outside of Sox9+ lineage is partially responsible for healing deficiencies in *Shh* cKO animals is still to be determined.

*Shh* appears to be particularly critical during the early stages of regeneration, as genetic deletion of *Shh* 5 days after injury still results in substantial healing (Fig. 3). This is further supported by our observation that deletion of *Smo* at 4 days after injury also results in complete healing (Fig. 4). In certain contexts, Hh signaling has been shown to be required for proliferation in the skeletal system^22^; however, we do not suspect this to be the case during large-scale bone regeneration, since in our previous work, we did not detect changes in proliferation or cell death when *Smo* is removed from Sox9+ lineage SSPCs during repair^7^. These observations, however, do not completely rule out a role for Hh signaling during later stages. For example, *Ihh* has an important role in chondrocytes and is required for their maturation in the growth plate^27^; so it is possible that Hh signaling could still have a role during later stages of regeneration by supporting chondrocyte maturation and differentiation in the repair callus. In addition, there is a growing body of evidence describing non-canonical, SMO-independent Hh signaling transduction (reviewed by Pietrobono et al., 2019^43^), which would allow for Hh ligand activity later during the repair process, even in *Smo* cKO contexts. Although evidence for SMO-independent Hh signaling is primarily observed in diseased contexts^42,43^, it would be interesting to investigate if similar mechanisms may be active during the late stages of normal bone healing.

We have proposed that Sox9+ lineage SSPCs act as “messenger” cells, since loss of *Smo* in this population (around 20% of the callus) also affects the behavior of Sox9-negative cells in the callus^7^. How cells outside the Sox9+ lineage participate in repair is still unclear. For example, SSPCs can be found in the growth plate, periosteum, and bone marrow compartments^14^. One possibility is that periosteal-derived Sox9+ cells, once activated by Hh signaling, facilitate the formation of a large bridging callus by recruiting other cells types to participate in repair. Our scRNAseq data illuminated a population of *Cxcl12*-expressing cells that is reduced in number within the repair callus of *Sox9*:CreER;*Smo*^flox/flox^ animals at 4 dpi. Since *Cxcl12*-expressing and Sox9+ lineage cell populations appear quite distinct (as we observed minimal overlap between the tdTomato and CXCL12 labels) the possibility that Hh activation of periosteal Sox9+ lineage cells stimulates their differentiation into a *Cxcl12*-high expressing population, similar as described during bone development, seems unlikely^32^. Thus, perhaps the role of Sox9+ cells in large-scale repair is to recruit these *Cxcl12*-expressing cells and stimulate their osteochondral differentiation. While *Cxcl12* expression has been observed in a number of cell types, including endothelial cells, periosteum, and cartilage (reviewed in Yellowley 2013^55^), interestingly, recent work from the Ono group has shown that *Cxcl12*-expressing bone marrow stromal cells can adopt an SSPC-like state in response to bone injury and subsequently contribute to cartilage and bone callus formation^13^. Linking this observation with our scRNAseq data suggests that the impaired large-scale healing observed in our models of Hh inhibition, may be due to the failed recruitment of *Cxcl12*-expressing bone marrow cells into the callus to participate in regeneration. The idea that periosteal and BM-derived cells may work in collaboration must be tested more thoroughly in the future, as it implies crosstalk between periosteal and BM-derived SSPCs and the appropriate recombinase mouse lines to investigate these relationships are much needed. Excitingly, the recent emergence of Dre-based genetically modified mice unlocks the possibility of simultaneously lineage tracing multiple cell populations in parallel^56,57^.

In summary, together our data point to a model of skeletal regeneration where *Shh* expression is uniquely required to stimulate cartilage callus formation following large-scale injury. This revelation may inform future therapeutic strategies, especially in circumstances where large-scale regeneration of skeletal elements is required, such as following traffic accidents, combat wounds, and segmental resection due to bone cancer removal.

## Methods

### Mice and animal housing

All procedures were approved by the University of Southern California Institutional Animal Care and Use Committee (Protocol #: 11256, 20639). We used the following mouse lines: *Sox9:*CreER2 (Sox9^tm1(cre/ERT2)Haak^)^58^, *Shh*^*flox/flox*^ (B6;129-*Shh*^*tm2Amc*^/J; JAX 004293), *R26R:*tdTomato (B6;129S6-*Gt(ROSA)26Sor*^*tm9(CAG-tdTomato)Hze*^/J; JAX 007905), *Smo*^*flox/flox*^ (*Smo*^*tm2Amc*^/J; JAX 004526), C57BL/6 J (JAX 000664), *Ihh:*LacZ (Ihh^tm1.1Bhum^, MGI:5316284)^26^, Cagg:CreER^(B6.Cg-Tg(CAG-cre/Esr1*)5Amc/J^, JAX 004682). All lines are listed in Table 1. Both male and female mice between 8–12 weeks old were used for experiments. Experimental *Shh* cKO mice crossed with Cagg:CreER carried either *Shh*^*Δ/*flox^ or *Shh*^*flox/flox*^ alleles. We did not detect any difference in outcome in these two genotypes. Control mice were a mix of uninduced and Cre positive, tamoxifen-induced heterozygotes, or tamoxifen treated Cre negative siblings. See each figure legend for details. Tamoxifen was injected intraperitoneally as 100 uL of a 20 mg/ml stock of tamoxifen (Sigma-Aldrich: T5648-1G, dissolved in corn oil at 60 °C for 2 h). When using the *Sox9*:CreER transgene target Sox9+ lineage cells, it is important to avoid targeting the potentially wide range of cells that may turn on *Sox9* in response to injury. To make sure that there is no residual tamoxifen in the animal’s system at the time of injury which would confound the experiment, tamoxifen is administered for several days prior to injury followed by a several-day chase until the injury assay. Since the Cagg:CreER is globally expressed, residual tamoxifen is not a concern and several days of injections leading up to the day of surgery were used to obtain maximum Cre recombination. After the DTR was induced in *Sox9*-expressing cells, using the tamoxifen inducible Cre, cell ablation was performed by injection of diphtheria toxin (1 μg per injection in 100 μl sterile H_2_0) 3 days prior to injury.

**Table 1:** List of reagents used in this study.

Reagent Type	Designation	Source/Reference	Identifiers	Additional Information
Genetic Reagent (M. musculus)	C57BL/6 J	JAX 000664	MGI:3028467	Bl6
Genetic Reagent (M. musculus)	*Sox9*^*tm1(cre/ERT2)Haak*^	Soeda et al., 2010^58^	MGI:	*Sox9:*CreER
			4867441	H Akiyama
Genetic Reagent (M. musculus)	*Smo*^*tm2Amc*^/J	JAX 004526	MGI:2176256	*Smo*^*flox/flox*^
Genetic Reagent (M. musculus)	Ihh^tm1.1Bhum^	Fabian et al., 2012^26^	MGI:5316284	*Ihh*:LacZ
				AP McMahon
Genetic Reagent (M. musculus)	B6.Cg-Tg(CAG-cre/Esr1*)5Amc/J	JAX 004682		Cagg:CreER
Genetic Reagent (M. musculus)	Gt(ROSA)26Sor^tm9(CAG-tdTomato)Hze^	JAX 007909	MGI:3809523	Ai9
Genetic Reagent (M. musculus)	C57BL/6-*Gt(ROSA)26Sor*^*tm1(HBEGF)Awai*^/J	Jax 007900		*R26:*DTR
Genetic Reagent (M. musculus)	B6;129-*Shh*^*tm2Amc*^/J	JAX 004293	MGI:1934268	*Shh*^*flox/flox*^
				AP McMahon
Antibody	Chicken polyclonal anti-mCherry	Novus Biological	NBP2-25158SS	1:200
Antibody	Rabbit polyclonal Anti-Sdf1(Cxcl12)	Abcam	ab25117	1:200
Antibody	Alexa Fluor 568 goat anti-rabbit	ThermoFisher	A-11011	1:500
Antibody	Alexa Fluor 568 goat anti-chicken	Abcam	ab175477	1:500

The reagents for this study and their sources are indicated.

### Injury assays

The following procedures were performed after inducing anesthesia with 2–4% isoflurane.

To perform rib resections, the surgical area overlying ribs 8–10 was prepared and 2 cm incision made through the skin, underlying muscle, and fat layers. These were held open by retraction (Fine Science Tools, 17004-05). An incision was made through the intercostal muscles overlying the desired section of rib with a 5.0 mm (Fine Science Tools, 10315-12) scalpel and the bone was separated from the muscle with fine tip forceps (Dumox #55). To create a resection while retaining the periosteum in the animal, the periosteum was incised along the length of rib with a 5.0 mm scalpel and separated from the underlying bone laterally with a fine tip forceps. To make a 3.0 mm resection, a cross-section was made through the bone at one end with fine micro-scissors (Fine Science Tools, 15000-04) and the bone was then lifted out of the periosteum and cut at the other end, 3 mm away. To close, the intercostal muscles were sutured over the top with 9–0 sutures (Ethicon, 2819G). The retractor was removed, and the overlying muscle and fat was also sutured with 9–0 sutures, followed by closing the skin with 7–0 sutures (Ethicon, 8700H). The incision was then secured with suture adhesive (Abbot, 32046–01)^6,7^. Surgical fractures were performed under identical opening conditions except that the rib bone was simply cut all the way through with surgical scissors in lieu of resecting a 3 mm segment. Post-operative pain was managed with subcutaneous buprenorphine SR (ZooPharm) at a dose of 0.5 uL/gram body weight.

To perform femur fractures, left hind limbs were prepared with a surgical field and a 3 mm incision was made medial to the patellar tendon^59^. The patella was dislocated laterally, and the femoral condyles exposed. A small hole was then drilled into the trochlear groove and a 26-gauge needle was inserted in retrograde fashion into the femoral intramedullary canal, stopping at the greater trochanter. The dislocated patella was reduced and sutured closed. Using a modified Bonnarens & Einhorn’s fracture apparatus^60^, a closed, mid-diaphyseal femoral fracture was then created. Radiographic images of the fractured femora were obtained immediately to verify production of a transverse, mid-diaphyseal fracture. Post-operative pain was managed with buprenorphine SR (ZooPharm) at a dose of 0.5 uL/gram body weight antibiotics through the drinking water for 5 days. The animals were allowed to bear weight immediately and to eat and drink ad libitum.

### Histology tissue processing

For paraffin embedded samples: Skeletal tissue along with the adjacent muscle and connective tissues was fixed with 4% PFA overnight at 4 °C, decalcified with 20% ETDA at pH 7.5 for 10–14 days, and then processed for paraffin embedding. A microtome (Shandon Finesse Me+: 77500102) was used to cut paraffin sections 8 microns thick. The sections were mounted on Superfrost Plus slides (VWR, 48311-703).

For cryo-embedded samples: Skeletal tissue along with the adjacent muscle and connective tissues was fixed in 4% PFA at room temperature for 30 min and placed in 30% sucrose overnight at 4 °C. The samples were embedded in OCT and flash frozen in an EtOH dry ice bath. 10 µM thick sections were cut using a Leica CM3050 S cryostat. Tape (cryofilm type 3 C(16UF) C-FUF303) was used to preserve the histology of the bone^61,62^. OCT was removed with a 1xPBS wash before mounting.

Hematoxylin and eosin (H&E), Safranin-O, and Xgal staining was carried out using standard protocols. Whole-mount samples were fixed in 95% EtOH overnight at 4 °C and alizarin red and alcian blue staining was performed as using a standard protocol^63^.

### Regeneration scoring

Regeneration success of each animal was scored using a semi-quantitative method as either poor, moderate, or good, based on a rubric that considers histological features similar to previously published (Table 2)^64^. “Poor” was judged unlikely to heal, a “Moderate” score indicated uncertainty in healing outcome, while “Good” was judged likely to exhibit successful regeneration. Samples were de-identified and each animal was scored by two judges. Animals from all time points are pooled together by genotype for graphical representation. If an animal was scored differently by the two judges, it was settled by a third judge.

**Table 2:** Rubric for qualitative scoring of healing.

	Poor	Moderate	Good
10 or 14 dpi	Minimal/no cartilage, and if cartilage does exist, it is located near cut ends and does not fill resection gap	Moderate cartilage production, incomplete bridging, appears unclear if injury will fully heal at extended time points	Substantial and thick cartilage production bridging the injury site
28 dpi	Little bone production. Injured bone end remains very disconnected	Moderate bone production. Some cartilage may still be present. Cuts end are not completely bridged, but appears full thickness healing is still possible	Injury site fully connected with thick newly generate bone

A rubric was used to define healing as Poor, Moderate, or Good.

### Quantification of skeletal repair

Skeletal repair could also be quantified to determine the degree of healing. Using a combination of H&E and Safranin-O stained histological sections, the amount of skeletal tissue as a percentage of total callus area, was quantified in ImageJ. In brief, using a mid-sagittal section, the total callus area (bounded by intercostal muscles and the resection sites) was defined as a region of interest. Within this region, cartilage area was identified by Safranin-O staining and bone was identified by trabecular structures vested with bone marrow. These areas were then measured and represented as a percentage of the total callus area. Animals at time points ranging from 7 to 28 dpi were pooled for graphical representation. For box and whiskers plots, the box represents the interquartile range and the horizontal line through the box represents the median. Whiskers extend to the maximum and minimum value. The data for each experiment was analyzed separately with a linear regression model comparing data from each genotype to the reference genotype for that experiment using the formula @formula(percentage ~ geno) where ‘percentage’ represents the percentage of the callus occupied by skeletal tissue and ‘geno’ is a categorical variable describing the genotype. The results and P values were calculated in Julia using a GLM.jl package.

### RNA in situ hybridization

Fluorescent RNA in situ hybridization (RNA-ISH) was performed on 8 µm paraffin sections. Following dewaxing in Xylene, and rehydration through an EtOH series, the tissue was permeabilized with Proteinase K (3.75ug/ 100 mls) and 1xPBS 0.1% Tween. Complementary DIG or FL labeled RNA probes were generated following kit instructions (Sigma-Aldrich: 11277073910 and 11685619910). After pre-hybridization for 1 h, probes (5 ng/ul in hybridization buffer) were overlayed on the sections overnight at 68 degrees followed by denaturing washes. Antigen blocking (0.5% Blocking buffer, Perkin Elmer) was then followed by antibody incubation using Anti-Digoxigenin-POD (Sigma-Aldrich: 11207733910) or Anti-Fluorescein-POD (Sigma-Aldrich: 11426346910) antibodies. Color detection was performed with TSA PLUS fluorescein or Cy3 (Akoya Biosciences, NEL753001KT). Slides with fluorescence were mounted with Vectashield with DAPI (Vector Laboratories: H1200) and were imaged with a Nikon AZ100 Macroscope and digital camera (Nikon Digital sight DS-Fi1). Fluorescent images were edited for contrast and color levels in Adobe Photoshop CS5.

Probes were made by RNA-transcription from plasmids containing 500–1000 bp gene fragments. The sequences used are listed in Table 3.

**Table 3:** Probes for RNA in situ hybridization.

Gene and ID	Nucleotides	Sequence ID	Citation
*Shh (Gene ID: 20423)*	300–942	NM 009170.3	Echelard et al.,1993^64^
*Ihh (Gene ID: 16147)*	1110–1684	NM 010544.3	Iwasaki et al.,1997^65^
*Dhh (Gene ID: 13363)*	263–1208	NM 007857.5	Bitgood and McMahon,1995^23^
*Prrx1* (*Gene ID: 18933)*	2486–2987	NM 001025570.1	Parrilla et al., 2016^66^

The sequences used to generate probes used in this study are indicated.

### Immunofluorescence

Antibody staining was performed on paraffin sections^7^. Paraffin sections, 8 µm, were dewaxed for 60 min in Citrisolv, rehydrated through and EtOH series, and permeabilized with 0.1% Triton-X prior to antigen retrieval with 10 mM sodium citrate, 0.05% Tween 20, pH of 6.0 in a 95 °C water bath for 30 min. After blocking with goat serum, antibody incubation was performed overnight at 4 °C. Primary antibodies used were anti-CXCL12, (Abcam: ab25117, 1:200); anti-mCHERRY, (Novus Biological: NBP2-25158SS, 1:200). After a series of washes to remove non-specific binding of the primary antibody, samples were incubated in secondary antibodies (Alexa Fluor 568 goat anti-chicken (Abcam: ab175477, 1:500), Alexa Fluor 568 goat anti-rabbit (ThermoFisher: A-11011, 1:500)). All antibodies used are listed in Table 1.

### scRNAseq preparation and analysis

Repair calluses were dissected from control (*Sox9*:CreER;*R26*:tdT;*Smo*^*flox/+*^) and *Sox9*:CreER;*R26*:tdT;*Smo*^*flox/flox*^ mice at 4 dpi. Care was taken to excise repair callus leaving the majority of adjacent skeletal muscle behind. Calluses were digested at 37 °C for 60 min on a shaker at 50 rpm in digest buffer (3 mg/mL Collagenase II (Worthington: LS004176), 1 U/mL Dispase (Corning: #354235), in α-MEM (STEMCELL Technologies: #36453)). Cells were then filtered through 40 micron filter tips (Sigma: BAH136800040), spun at 300 g for 5 min, and resuspended for dead cell removal using the Dead Cell Removal Kit (Miltenyi Biotec: #130-090-101). Cells were then resuspended in 1xPBS for loading into the 10x Chromium platform. Six calluses were dissected, digested, and pooled by genotype (3 animals for each genotype) for generation of single-cell libraries. Libraries were sequenced on an Illumina NextSeq machine.

Reads were aligned to the mm10 genome using 10x Genomics’ Cell Ranger pipeline (v3.0.2) with default parameters. Cell Ranger outputs were used for downstream analysis in Seurat (v4.05). QC, clustering, and analysis was performed using the code shown in Supplementary Methods. Cells with <200 or >2000 unique genes were excluded from analysis as they may represent poor quality cells or doublets. Cells with >10% of total reads mapping to mitochondrial genes (defined as any gene beginning with “Mt-”) were also excluded from downstream analysis. Clustering was done using Louvain method at resolution of 0.4. We used UMAP dimensional reduction to visualize clusters and Wilcoxon Ran Sum test to identify differentially expressed genes. Datasets from this analysis can be found at GEO GSE196060.

### Reporting summary

Further information on research design is available in the Nature Research Reporting Summary linked to this article.

## Supplementary information

Supplementary Information

REPORTING SUMMARY

## Data Availability

The datasets generated during and/or analyzed during the current study are available in the GEO repository, Accession #: GSE196060.

## Code availability

Cell Ranger (v3.0.2) with default parameters was followed by analysis in Seurat (v4.05). The code used to generate the results can be found in Supplementary Methods.
