# Supplementary material for: A murine model of large-scale bone regeneration reveals a selective requirement for Sonic Hedgehog
Source: NPJ Regen Med. 2022 May 17;7:30. doi: 10.1038/s41536-022-00225-8 (PMC9114339; doi:10.1038/s41536-022-00225-8)
Supplement: Supplementary file 1 — Supplementary Information [file 41536_2022_225_MOESM1_ESM.pdf]

Supplementary Figure 1

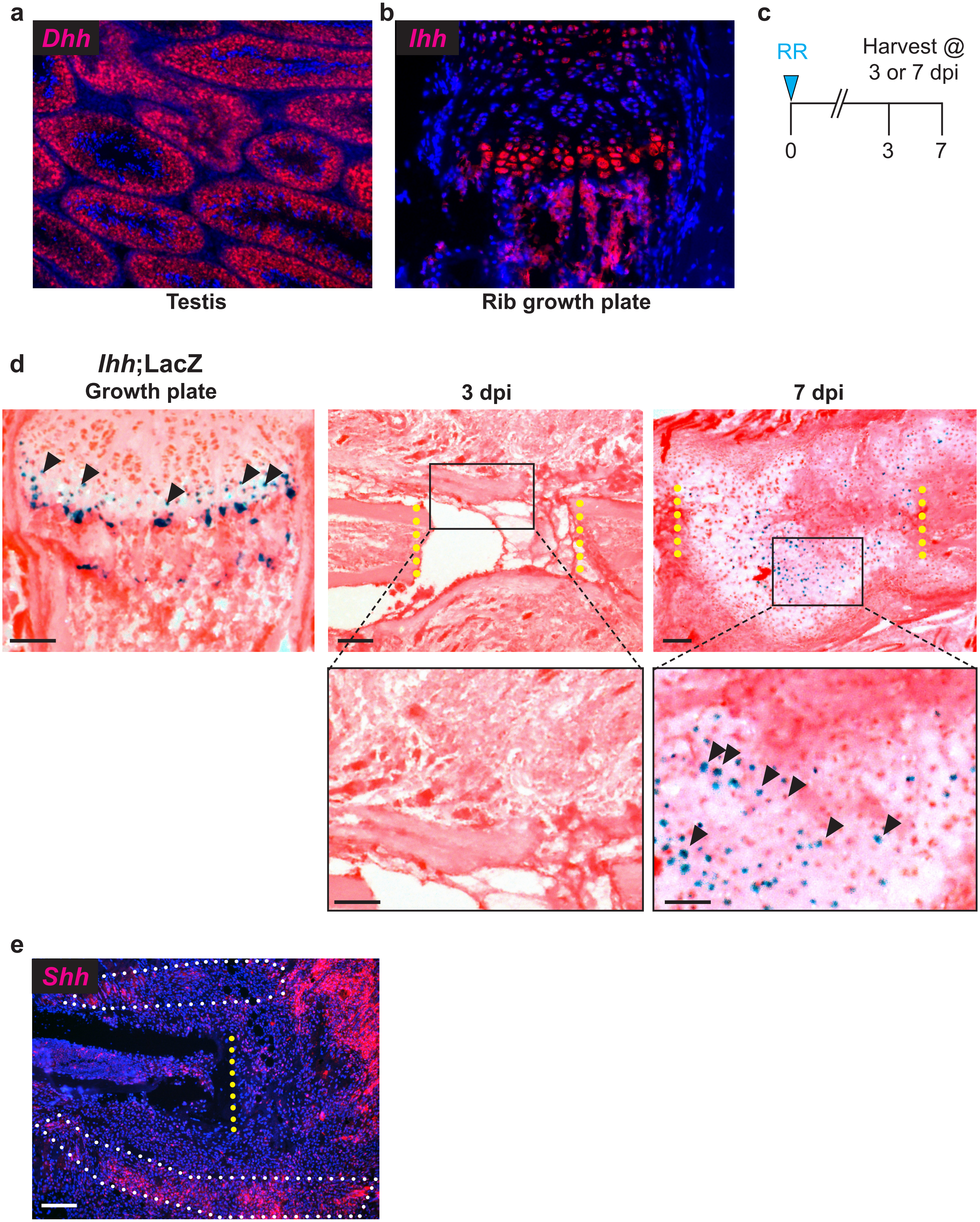

Supplementary Figure 1.
***Ihh* is upregulated later in mature chondrocytes during callus differentiation.**

(a) RNA-ISH for *Dhh* showing the expected expression pattern in murine testis tissue.

(b) RNA-ISH for *Ihh* showing the expected expression in rib growth plate chondrocytes.

(c) *Ihh;*LacZ mice have a LacZ reporter cassette knocked into the first exon of the endogenous *Ihh* gene, allowing LacZ expression to be used as a readout of *Ihh* expression. Rib resections were performed at day 0 and tissues were harvested at 3 or 7 dpi.

(d) As expected, LacZ expression is detectable with Xgal staining in mature chondrocytes of the growth plate (black arrowheads) from uninjured samples. In injured samples, LacZ expression is not detectable at 3 dpi. At 7 dpi, LacZ expression can be detected in many large, mature chondrocytes in regions of the callus that have begun differentiating (black arrowheads). Surgical cut sites are indicated with yellow dotted lines. N = 2. Scale bars = 100 microns

(e) RNA-ISH for *Shh* at higher magnification highlighting low expression in the cells found near the periphery of the callus.

Supplementary Figure 2

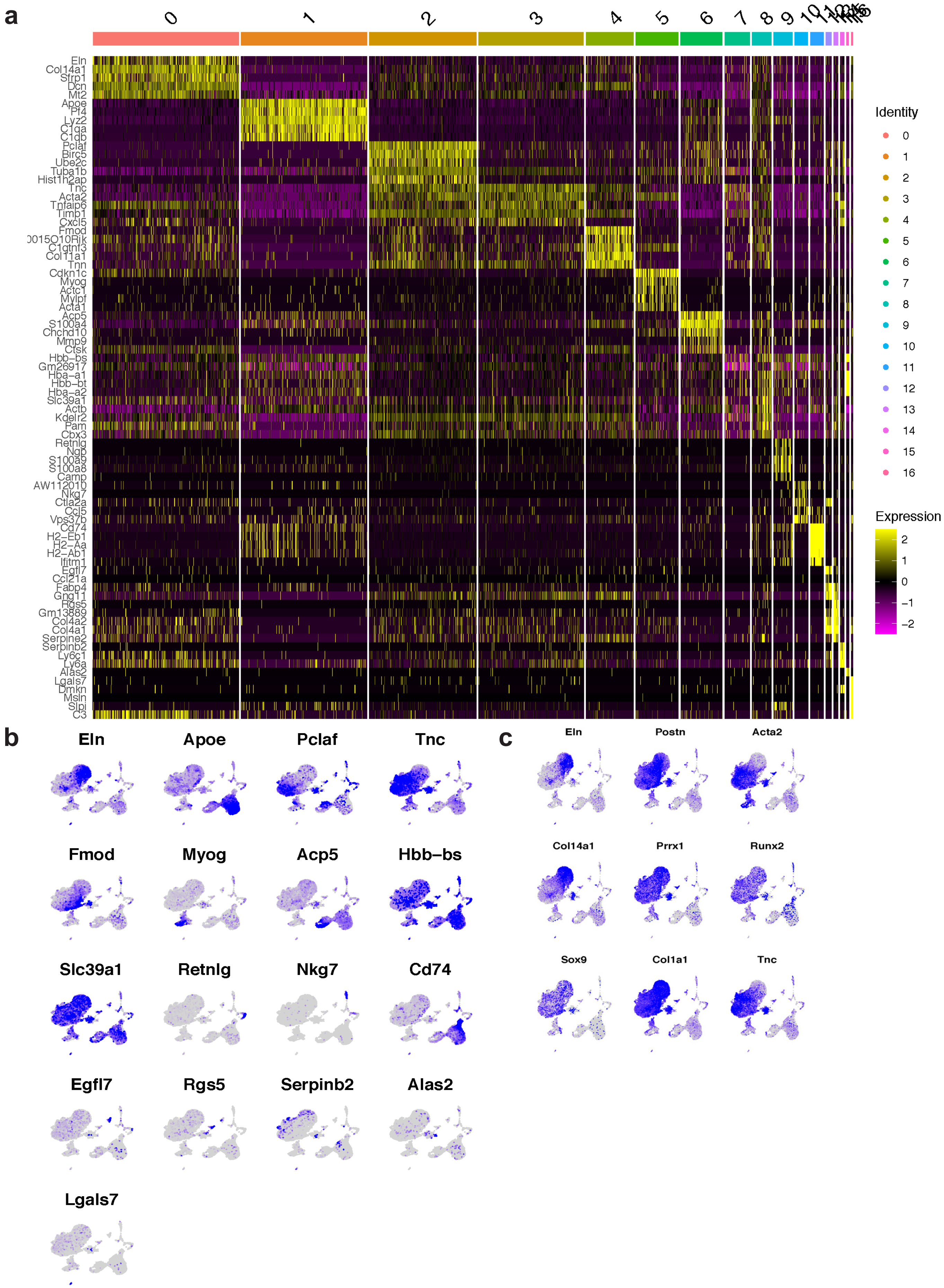

Supplementary Figure 2.
Characterization of scRNAseq clusters

(a) Heat map showing the expression the top 5 marker genes for all 17 clusters identified from Figure 9C.

(b) Feature plot showing the expression of a top marker gene for each of 17 clusters identified in Figure 9C.

(c) Feature plot showing the expression of 9 marker genes used to identify the large cluster as "connective tissue" for further analysis in Figure 9E.

Supplementary Methods

Code used for scSEQ analysis:
Cell Ranger (v3.0.2) was used with default parameters, followed by analysis in Seurat (v4.05):

```
ctrl.data <- Read10X(data.dir =
"~/Desktop/4dpr_control_filtered_feature_bc_matrix_new/")
KO.data <- Read10X(data.dir =
"~/Desktop/4dpr_KO_filtered_feature_bc_matrix_new/")

# Set up control object
ctrl <- CreateSeuratObject(counts = ctrl.data, project = "4DPR_CTRL",
min.cells = 3, min.features = 200)
ctrl$gt <- "CTRL"

# Set up stimulated object
KO <- CreateSeuratObject(counts = KO.data, project = "4DPR_KO", min.cells
= 3, min.features = 200)
KO$gt <- "KO"

# Combine Seurat objects
ctrlKO.combined <- merge(ctrl, y = KO, project = "4dprcombined")

# Standard QC
ctrlKO.combined[["percent.mt"]] <- PercentageFeatureSet(ctrlKO.combined,
pattern = "^mt-")

VlnPlot(ctrlKO.combined, features = c("nFeature_RNA", "nCount_RNA",
"percent.mt"), ncol = 3, pt.size = 0.001)

plot1 <- FeatureScatter(ctrlKO.combined, feature1 = "nCount_RNA",
feature2 = "percent.mt")
plot2 <- FeatureScatter(ctrlKO.combined, feature1 = "nCount_RNA",
feature2 = "nFeature_RNA")
CombinePlots(plots = list(plot1, plot2))

ctrlKO.combined <- subset(ctrlKO.combined, subset = nFeature_RNA > 200 &
nFeature_RNA < 6000 & percent.mt < 10)

ctrlKO.combined <- NormalizeData(ctrlKO.combined, normalization.method =
"LogNormalize", scale.factor = 10000)

ctrlKO.combined <- FindVariableFeatures(ctrlKO.combined, selection.method
= "vst", nfeatures = 2000)

all.genes <- rownames(ctrlKO.combined)
ctrlKO.combined <- ScaleData(ctrlKO.combined, features = all.genes)

# Dimensionality Reduction
ctrlKO.combined <- RunPCA(ctrlKO.combined, features =
VariableFeatures(object = ctrlKO.combined))

ElbowPlot(ctrlKO.combined, ndims = 30)
```

```
# Clustering
ctrlKO.combined <- FindNeighbors(ctrlKO.combined, dims = 1:20)
ctrlKO.combined <- FindClusters(ctrlKO.combined, resolution = 0.3)

ctrlKO.combined <- RunUMAP(ctrlKO.combined, dims = 1:20)

DimPlot(ctrlKO.combined, reduction = "umap", split.by = "gt")

ctrlKO.combined.markers <- FindAllMarkers(ctrlKO.combined, only.pos =
TRUE, min.pct = 0.25, logfc.threshold = 0.5)
ctrlKO.combined.markers %>% group_by(cluster) %>% top_n(n = 2, wt =
avg_logFC)

# Find your gene!
FeaturePlot(ctrlKO.combined, features = "Cxcl12", order = T, split.by =
"gt")

# Find top5 markers per cluster
ctrlKO.combined.markers %>%
  group_by(cluster) %>%
  top_n(n = 5, wt = avg_log2FC) -> top5

DoHeatmap(ctrlKO.combined, features = top5$gene)
```
