# Supplementary material for: A murine model of large-scale bone regeneration reveals a selective requirement for Sonic Hedgehog
Source: NPJ Regen Med. 2022 May 17;7:30. doi: 10.1038/s41536-022-00225-8 (PMC9114339; doi:10.1038/s41536-022-00225-8)
Supplement: Supplementary file 2 — REPORTING SUMMARY [file 41536_2022_225_MOESM2_ESM.pdf]

nature portfolio

Corresponding author(s): Francesca Mariani

Last updated by author(s): Feb 10, 2022

Reporting Summary

Nature Portfolio wishes to improve the reproducibility of the work that we publish. This form provides structure for consistency and transparency in reporting. For further information on Nature Portfolio policies, see our Editorial Policies and the Editorial Policy Checklist.

Statistics

For all statistical analyses, confirm that the following items are present in the figure legend, table legend, main text, or Methods section.

n/a	Confirmed	
☐	☒	The exact sample size (n) for each experimental group/condition, given as a discrete number and unit of measurement
☐	☒	A statement on whether measurements were taken from distinct samples or whether the same sample was measured repeatedly
☐	☒	The statistical test(s) used AND whether they are one- or two-sided<br>*Only common tests should be described solely by name; describe more complex techniques in the Methods section.*
☐	☒	A description of all covariates tested
☒	☐	A description of any assumptions or corrections, such as tests of normality and adjustment for multiple comparisons
☐	☒	A full description of the statistical parameters including central tendency (e.g. means) or other basic estimates (e.g. regression coefficient) AND variation (e.g. standard deviation) or associated estimates of uncertainty (e.g. confidence intervals)
☒	☐	For null hypothesis testing, the test statistic (e.g. F, t, r) with confidence intervals, effect sizes, degrees of freedom and P value noted<br>*Give P values as exact values whenever suitable.*
☒	☐	For Bayesian analysis, information on the choice of priors and Markov chain Monte Carlo settings
☒	☐	For hierarchical and complex designs, identification of the appropriate level for tests and full reporting of outcomes
☐	☒	Estimates of effect sizes (e.g. Cohen's d, Pearson's r), indicating how they were calculated

Our web collection on statistics for biologists contains articles on many of the points above.

Software and code

Policy information about availability of computer code

Data collection	none
Data analysis	CellRanger v3.0.2, Seurat v4.0.5

For manuscripts utilizing custom algorithms or software that are central to the research but not yet described in published literature, software must be made available to editors and reviewers. We strongly encourage code deposition in a community repository (e.g. GitHub). See the Nature Portfolio guidelines for submitting code & software for further information.

Data

Policy information about availability of data

All manuscripts must include a data availability statement. This statement should provide the following information, where applicable:

- Accession codes, unique identifiers, or web links for publicly available datasets
- A description of any restrictions on data availability
- For clinical datasets or third party data, please ensure that the statement adheres to our policy

Datasets from scRNAseq analysis can be found at GEO: GSE196060. Upload is complete, and public as of 2/6/2022.

Field-specific reporting

Please select the one below that is the best fit for your research. If you are not sure, read the appropriate sections before making your selection.

☒ Life sciences ☐ Behavioural & social sciences ☐ Ecological, evolutionary & environmental sciences

For a reference copy of the document with all sections, see nature.com/documents/nr-reporting-summary-flat.pdf

Life sciences study design

All studies must disclose on these points even when the disclosure is negative.

Sample size	Power calculations were performed using preliminary data to estimate effect size and calculate sample sizes.
Data exclusions	No data were exluded
Replication	For scSEQ, one run was performed for each genotype but based on 3 biological replicates per genotype (stated in Methods). All other experiments were repeated and the biological replicates (N) are stated. In addition, variation in groups is made transparent with both qualitative and quantitative scoring.
Randomization	Male and female mice from each litter were randomized into groups.
Blinding	When possible, investigators were blinded to the genotype and treatment regimen during all experimental procedures and data analyses.

Reporting for specific materials, systems and methods

We require information from authors about some types of materials, experimental systems and methods used in many studies. Here, indicate whether each material, system or method listed is relevant to your study. If you are not sure if a list item applies to your research, read the appropriate section before selecting a response.

Materials & experimental systems

n/a	Involved in the study	
☐	☒	Antibodies
☒	☐	Eukaryotic cell lines
☒	☐	Palaeontology and archaeology
☐	☒	Animals and other organisms
☒	☐	Human research participants
☒	☐	Clinical data
☒	☐	Dual use research of concern

Methods

n/a	Involved in the study	
☒	☐	ChIP-seq
☒	☐	Flow cytometry
☒	☐	MRI-based neuroimaging

Antibodies

Antibodies used	anti-CXCL12, (Abcam: ab25117); anti-mCHERRY, (Novus Biological: NBP2-25158SS), Alexa Fluor 568 goat anti-chicken (Abcam: ab175477), Alexa Fluor 568 goat anti-rabbit (ThermoFisher: A-11011).
Validation	All antibodies are commercially available, widely used, and extensively validated in the literature.

Animals and other organisms

Policy information about studies involving animals; ARRIVE guidelines recommended for reporting animal research

Laboratory animals	Sox9:CreER2 (Sox9tm1(cre/ERT2)Haak), Shhflox/flox (B6;129-Shhtm2Amc/J; JAX 004293), R26R:tdTomato (B6;129S6-Gt(ROSA)26Sortm9(CAG-tdTomato)Hze/J; JAX 007905), Smoflox/flox (Smotm2Amc/J; JAX 004526), C57BL/6J (JAX 000664), Ihh:LacZ (Ihhtm1.1Bhum, MGI:5316284), Cagg:CreER (B6.Cg-Tg(CAG-cre/Esr1*)5Amc/J, JAX 004682). Both male and female mice between 8–12 weeks old were used for experiments.
Wild animals	None
Field-collected samples	None
Ethics oversight	All animal procedures were approved by the University of Southern California Institutional Animal Care and Use Committee (Protocol #: 11256, 20639).

Note that full information on the approval of the study protocol must also be provided in the manuscript.
